# Pharmacist-led primary care interventions to promote medicines optimisation and reduce overprescribing: a systematic review of UK studies and initiatives

**DOI:** 10.1136/bmjopen-2023-081934

**Published:** 2024-08-07

**Authors:** Duncan Chambers, Louise Preston, Mark Clowes, Anna J Cantrell, Elizabeth C Goyder

**Affiliations:** 1 Sheffield Centre for Health and Related Research (SCHARR), School of Medicine and Population Health, University of Sheffield, Sheffield, UK

**Keywords:** primary care, quality in health care, patient-centered care, systematic review

## Abstract

**Abstract:**

**Objectives:**

To systematically review and synthesise evidence on the effectiveness and implementation barriers/facilitators of pharmacist-led interventions to promote medicines optimisation and reduce overprescribing in UK primary care.

**Design:**

Systematic review.

**Setting:**

UK primary care.

**Methods:**

We searched MEDLINE, Embase, CINAHL PsycINFO and The Cochrane Library for UK-based studies published between January 2013 and February 2023. Targeted searches for grey literature were conducted in May 2023. Quantitative and qualitative studies (including conference abstracts and grey literature) that addressed a relevant intervention and reported a primary outcome related to changes in prescribing were eligible for inclusion. Quality of included studies was assessed using the Multiple Methods Appraisal Tool. We performed a narrative synthesis, grouping studies by publication status, setting and type of data reported (effectiveness or implementation).

**Results:**

We included 14 peer-reviewed journal articles and 11 conference abstracts, together with 4 case study reports. The journal articles reported 10 different interventions, 5 delivered in general practice, 4 in care homes and 1 in community pharmacy. The quality of evidence was higher in general practice than in care home settings. It was consistently reported that the intervention improved outcomes related to prescribing, although the limited number of studies and wide range of outcomes reported made it difficult to estimate the size of any effect. Implementation was strongly influenced by relationships between pharmacists and other health and care professionals, especially general practitioners. Implementation in care homes appeared to be more complex than in general practice because of differences in systems and ‘culture’ between health and social care.

**Conclusions:**

Pharmacist-led interventions have been reported to reduce overprescribing in primary care settings in the UK but a shortage of high-quality evidence means that more rigorous studies using high-quality designs are needed. More research is also needed in community pharmacy settings; to assess intervention effects on patient outcomes other than prescribing and to investigate how reducing overprescribing can impact health inequalities.

**PROSPERO registration number:**

CRD42023396366.https://fundingawards.nihr.ac.uk/award/NIHR135767

**STRENGTHS AND LIMITATIONS OF THIS STUDY**

- We included evidence often excluded from systematic reviews to get as full a picture as possible of how pharmacist-led interventions are implemented and sustained in practice as well as their characteristics and effectiveness.
- Many of the studies lacked a control group and the research took place in a highly complex and evolving system, meaning that results could have been influenced by confounding factors such as other interventions in the health and social care system.
- Some review processes were performed by a single reviewer and meta-analysis was not feasible.

## Introduction

 This evidence review was performed to support implementation of the National Overprescribing Review for England (NOR; see below)) by examining research on pharmacist-led overprescribing interventions in UK primary care settings. Pharmacists are trained to provide advice and support to patients and other health professionals, pharmacist-independent prescribers (PIPs) have existed since 2006 and patients are increasingly asked to consider the community pharmacy as a first source of support for minor health conditions. Alongside community pharmacies, many general practices have pharmacists as members of the practice team. Pharmacists, working with general practitioners (GPs) and other healthcare professionals, are thus well placed to support interventions directed towards medicines optimisation and the reduction of overprescribing. Such interventions include carrying out structured medication reviews (SMRs) directly with patients and carers and/or reviewing data from patient records. The aims and objectives of the review are outlined below, following a brief clarification of terminology.

Overprescribing has been defined as ‘the use of a medicine where there is a better non-medicine alternative, or the use is inappropriate for that patients’ circumstances and wishes’.^1^ Overprescribing is often related to the concept of problematic polypharmacy, where harmful effects result from the prescription of multiple medications. However, there is no agreed definition of polypharmacy and patients with complex health conditions may require multiple medications.

Medicines optimisation is an umbrella term for interventions designed to ensure that medicines are used safely and effectively, producing the best possible outcomes for patients. In this context, deprescribing refers to the process of stopping medications that are no longer appropriate to a patient’s needs. Deprescribing is a response to overprescribing and problematic polypharmacy and involves collaboration between health professionals and patients and/or carers to ensure shared decision-making. Shared decision-making with patients and/or carers is fundamental to successful medicines optimisation^2^ but the need for time and resources to ensure that this takes place can create barriers to service delivery. Another related term, medicines reconciliation, is a more technical process to ensure consistency between prescription records and the medications the patient is actually receiving and taking. The terminology around overprescribing and other forms of medicines misuse was recently reviewed by Singier *et al*.^3^ Medication review involves examining a patient’s prescriptions as a whole and is separate from measures to reduce inappropriate prescribing of specific medications or types of medication such as antibiotics or proton pump inhibitors.

Overprescribing can cause direct harm to patients in a variety of ways. It has been estimated that about 6.5% of hospital admissions are caused by harmful effects of medication, rising to 20% for people aged over 65.^1^ In addition to physiological harms, long-term use of some medications can lead to dependency and problems when attempting to withdraw the medication.

Issues relating to prescribed medication can arise from a whole range of causes, including patients requiring treatment for multiple conditions, lack of coordination between different health professionals or organisations and failures of communication between health professionals and patients (eg, failing to gather information because of time constraints on appointments). Availability of new medications and increasing numbers of people living with long-term conditions such as arthritis and diabetes have resulted in patients being prescribed more medications and continuing to take them for long periods of time, often for life. The average number of prescription items per head of population doubled between 1996 and 2016, and over 75% of prescriptions are repeat prescriptions.^1^

Pharmacists are thus well placed to support processes of medicines optimisation, which involve them working closely with medical professionals (particularly GPs), commissioners of healthcare and patients. The report of the NOR for England, published in 2021, provides numerous examples and case studies.^1^

The NOR for England was set up in 2018 to evaluate the extent of overprescribing in the National Health Service (NHS) and recommend measures to reduce it, particularly in primary care. A review of existing research (overview of systematic reviews) was commissioned to support the national review.^4^ The NOR identified a need for a more consistent and effective approach to medication review, which requires both the identification of effective interventions and an understanding of the factors that need to be addressed in terms of organisational and cultural barriers to implementation. The national review’s recommendations included changes to systems (patient records, transfers of care and clinical guidance) and culture (reduced dependence on medication and support for shared decision-making), as well as the appointment of a National Clinical Director for Prescribing.^1^

This evidence review was commissioned to support implementation of the NOR recommendations by examining research on pharmacist-led overprescribing interventions in UK primary care settings. Our focus on pharmacist-led interventions complements recent research on deprescribing in the UK context. The TAILOR evidence synthesis sought to identify how best to support deprescribing in older people living with multimorbidity and polypharmacy. The authors concluded that effective deprescribing requires ‘attention to providing an enabling infrastructure, access to data, tailored explanations and trust’.^5^ More recently, Radcliffe *et al* conducted a realist review and synthesis examining multidisciplinary medication review and deprescribing interventions for older people in primary care.^6^ This study identified a number of key mechanisms that could contribute to the design of effective interventions, including integration of pharmacists into the multidisciplinary team delivering the intervention. Pharmacist-led interventions could fall within the scope of both of these studies, but characterisation of the evidence base is required to support the application of insights derived from these more general, theory-based reviews.

We aimed to assess the effects of relevant interventions on outcomes related to prescribing, identify key characteristics of the interventions and examine barriers and facilitators to implementation in routine practice. A further aim was to assess the quality of the evidence base and identify priorities for further research.

## Methods

### Review aims and objectives

We aimed to perform a systematic review of published literature and published or informally published evaluations reporting UK-based, pharmacist-led interventions for overprescribing, including the following components:

1. A review and synthesis of outcomes of effective interventions.
2. A review of the characteristics of effective interventions using the Template for Intervention Description and Replication (TIDieR) framework.
3. Evaluation of the UK evidence base in terms of quality and risk of bias.
4. Identification of case study examples of effectively implemented interventions in the UK.

### Inclusion and exclusion criteria

Inclusion criteria for the review were as follows

- Population/setting: UK primary care.
- Intervention: Pharmacist-led interventions aimed at review and optimisation of prescribed medications.
- Comparator: Not required.
- Outcomes: Studies had to report a primary outcome related to changes in prescribing. Secondary outcomes were other patient and health service outcomes, including but not limited to changes to type of medicines prescribed, quality of life, hospital admissions and deaths.
- Study design: Quantitative and qualitative studies were eligible for inclusion, with no exclusions based on study design or quality. Reports of local initiatives published as grey literature reports or conference abstracts were included to give a fuller picture of activity across the NHS.
- Other: Studies published in English between January 2013 and February 2023.

We excluded interventions aimed at reducing overprescribing of specific medications or types of medication, for example, antibiotics or proton pump inhibitors. Studies of children and young people were also excluded.

### Search methods

The literature search harnessed economies of scale by identifying primary studies for inclusion in this review and reviews for inclusion in a scoping review for internal use to inform the wider project. Searches were conducted by an information specialist (MC) in order to identify published and unpublished evidence on primary care interventions to reduce overprescribing.

#### Phase 1: peer-reviewed literature

A first phase of database searches was run in February 2023 to retrieve relevant peer-reviewed literature. Searches were designed around the concepts mentioned in table 1.

**Table 1:** Key search concepts

Problem	Intervention	Setting
Overprescribing;Inappropriate prescribing; polypharmacy	Deprescribing;Structured medication review; medication reconciliation; medicines optimisation; shared decision making; personalised care	Primary care(including international terms for primary care where relevant)

While we are aware of the Morel filter (2022) for identifying studies of deprescribing,^7^ our focus was specifically on a primary care setting. Search strategies are provided in online supplemental file 1.

Searches covered the databases MEDLINE, Embase, CINAHL, PsycINFO and The Cochrane Library and were limited to studies published since 2013 and in OECD countries with healthcare systems similar to the UK.

#### Phase 2: grey literature

A further phase of targeted searches was conducted in May 2023 to identify unpublished or ‘grey’ literature. This involved searching for the case studies identified by the NOR (to identify any which had produced a report or evaluation), and then searching the Overton.io platform for pharmacist-led deprescribing/overprescribing and medicines optimisation.

Searches were complemented by input from stakeholders (internal and external topic advisers) to minimise the risk of missing any other relevant evidence.

### Study selection

Records retrieved by the literature search were stored in a shared EndNote library and deduplicated. Screening for inclusion at the title level was performed by single reviewers after piloting a test set. Reviewers could refer records to another team member in the event of uncertainty and a 20% sample of records was screened by a second reviewer to validate title-level inclusion decisions.

Screening for inclusion at the abstract and full-text level was performed by pairs of reviewers acting independently. Disagreements were resolved by discussion among the reviewers involved (AJC, DC and LP). A good level of agreement was achieved, values of kappa between pairs of reviewers ranging from 0.67 to 0.96. Reasons for exclusion at the full-text stage were recorded.

### Data extraction

Data extraction tables and summary tables were developed in Microsoft Word. Extraction was performed by a single reviewer, with a 10% sample being checked for consistency and accuracy. In addition to standard data extraction fields (study design/sample size, setting, intervention, key findings and strengths/limitations), we used the TIDieR Lite framework to collect information on the features of interventions reported as ‘successful’ to determine whether service commissioners and providers should consider specific factors when commissioning/delivering services. TIDieR Lite is a simplified version of the TIDieR checklist.^8^

### Quality assessment

Methodological quality of peer-reviewed journal articles was assessed using the Mixed Methods Appraisal Tool (MMAT) version 2018.^9^ The tool includes screening questions and methodological quality questions for different study designs (qualitative, randomised trials, non-randomised quantitative studies, descriptive studies and mixed methods). Quality assessment results were combined with identified strengths and limitations (including those reported by study authors) to characterise the contribution of individual studies and groups of studies to the overall evidence base.

### Data synthesis

We performed a narrative synthesis of the included studies using text and tables to describe study and intervention characteristics in line with methodological and reporting guidelines.^10 11^ We initially grouped studies by publication status, considering peer-reviewed journal articles (regardless of study design and quality) separately from conference abstracts and case studies. Within these three categories, we grouped studies by setting (general practice, care homes or community settings). We also distinguished between studies reporting effectiveness of interventions and those reporting implementation of interventions (eg, qualitative studies and process evaluations). In view of study heterogeneity and reporting limitations, effectively implemented interventions were defined as those where the study authors’ conclusions indicated that the service was regarded as a success and was planned to continue or be expanded.

Studies reported a wide variety of outcomes using diverse effect measures. For this reason, we did not attempt to calculate a standardised metric to compare effect sizes across outcomes. The synthesis used a ‘vote-counting’ method (number and proportion of studies reporting positive, negative or neutral outcomes), prioritising prescribing-related outcomes over patient and other outcomes. Reported effect measures and associated 95% CIs were recorded in the text and tables. Tables of study characteristics and findings were presented alphabetically by author for consistency. While reporting results from all study designs, we prioritised stronger study designs (experimental and quasi-experimental) over those of uncontrolled observational studies. In terms of exploring heterogeneity, the structure of the synthesis allowed consideration of potential modifiers including study design, study quality and setting. Intervention components and aspects of implementation were examined using modifications of existing frameworks, the component analysis was prespecified in the review protocol.

We did not use the Grading of Recommendations Assessment, Development and Evaluation approach to assess certainty of evidence because of its emphasis on randomised trials and downgrading of other study designs. Instead, we distinguished between controlled and uncontrolled studies, identified areas of consistency and inconsistency and highlighted areas of particularly limited evidence (eg, settings or outcomes represented by single studies). A similar approach has been used by team members in previous reviews.^12^

### Public involvement

The review was supported by a public panel that provided feedback on public perceptions that informed the review and are reflected in the Discussion.

### Variations from protocol

We used Tidier Lite instead of the full TIDieR framework. This was because the full framework is designed to allow the replication of interventions and therefore goes beyond the degree of detail required for evidence synthesis. The scoping review of reviews referred to in the protocol was not completed (see ‘Search methods’ above).

## Results

### Results of literature search

**Figure 1:**
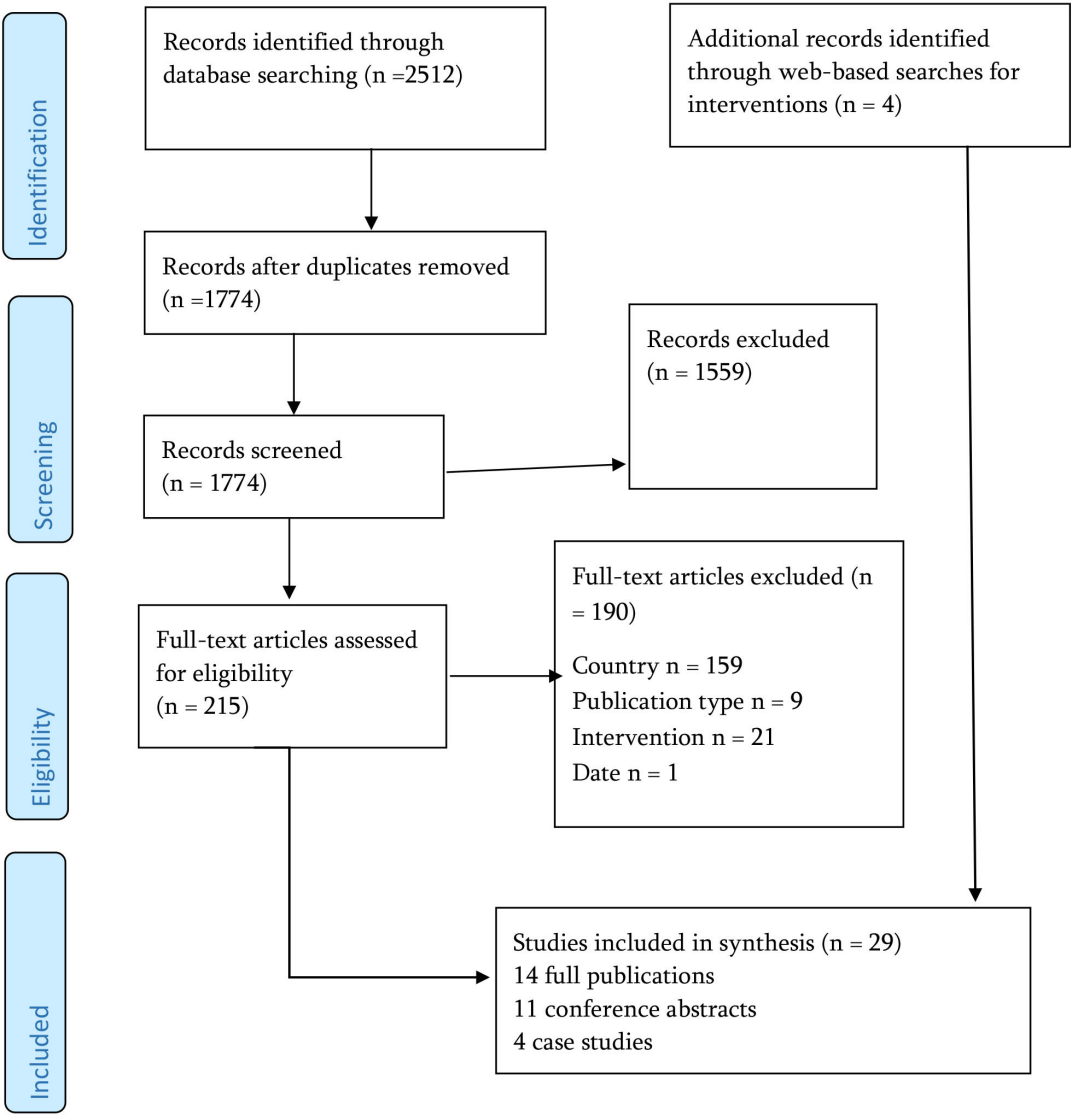
PRISMA flow diagram. PRISMA, Preferred Reporting Items for Systematic Reviews and Meta-Analyses.

The Preferred Reporting Items for Systematic Reviews and Meta-Analyses flow diagram (figure 1) summarises the study selection process. After screening 1774 records at the title and abstract stage and 215 full-text articles, we included 14 published articles, 11 conference abstracts and 4 case study reports. The majority of exclusions were of studies conducted outside the UK, with a smaller number excluded because the intervention was not pharmacist led or the article did not report empirical data. Characteristics of the included studies are reported in the following sections.

### Research studies

#### Study characteristics

Study characteristics are summarised in table 2, with full data extraction tables in online supplemental file 2. The 14 publications reported on 10 interventions, of which 5 were delivered in general practice (7 publications^13^,^19^), 3 in care homes for older people (5e publications^20^,^24^), 1 in care homes for people with intellectual disabilities (IDs)^25^ and 1 in community pharmacies.^26^

**Table 2:** Summary of research study characteristics

Reference	Population	Intervention	Study design	Outcome measures
Quantitative controlled studies				
Howard 2014^13^	Pharmacists delivering intervention	IT-enabled pharmacist-led review to reduce medication errors	Cluster RCT (PINCER trial)	Time taken to complete reviews; recommended interventions and whether they were implemented
Peek 2020^17^	General practice patients with one or more risk factors for hazardous prescribing or inadequate blood test monitoring	Pharmacist-led Safety Medication dASHboard (SMASH) intervention	Interrupted time series analysis	Rates (prevalence) of potentially hazardous prescribing and inadequate blood-test monitoring
Rodgers 2022^18^	General practices in the East Midlands	Pharmacist-led IT intervention (PINCER)	Multiple interrupted time series	Indicators of potentially hazardous prescribing
Syafhan 2021^19^	Patients in participating GP practices at risk of MRPs	Pharmacist-supplemented care focusing on medication optimisation	Individual RCT	Number of medication-related problems (MRPs) and medication inappropriateness plus clinical outcomes and costs
Quantitative uncontrolled studies				
Alves 2019^21^	Care home residents	Medication review by primary care pharmacists linked to GP practices	Service evaluation (5-year uncontrolled study)	Interventions by pharmacist (including deprescribing and changes to prescriptions)
Baqir 2017^22^	Care home residents	Medication review by pharmacist with or without GP	Retrospective analysis of data from QI programme	Number and type of medications stopped
Thayer 2021^25^	Care home residents with intellectual disabilities	Collaborative service initiative involving community pharmacists and a specialist mental health pharmacist providing review of medicines and lifestyle risk factors	Service evaluation	Pharmacist interventions /recommendations and acceptance by GPs and psychiatrists
Twigg 2015^26^	Patients over 65 prescribed four or more medications	Community pharmacist consultation including medication review using STOPP/START rules	Service evaluation	Number of recommendations; falls, medication adherence, quality of life and costs at 6 months
Qualitative/mixed methods				
Alharthi 2023^20^	Care home residents	Deprescribing by pharmacist-independent prescriber	Qualitative interviews with participants in a cluster RCT (CHIPPS study)	Barriers and facilitators to deprescribing
Birt 2021^23^	Care home residents	Pharmacist-independent prescribers responsible for medicines management (CHIPPS)	Mixed methods process evaluation	PIP activities, perceived benefits and barriers to implementation
Jeffries 2018^14^	Pharmacists delivering intervention, GPs and CCG staff	Pharmacist-led intervention involving the use of an electronic audit and feedback surveillance dashboard to identify patients potentially at risk of hazardous prescribing or monitoring of medicines in general practice	Qualitative interviews	Themes related to implementation of the intervention and role of practice pharmacists and others
Jeffries 2017^15^	Stakeholders in general practice and CCG	Electronic medicines optimisation system	Qualitative realist evaluation	Suggestions to support implementation of the system
Lane 2020^24^	Doctors, pharmacists, care-home managers and staff, residents and relatives	Pharmacist-independent prescriber service	Qualitative focus groups and interviews	Perceived benefits of the service and barriers and facilitators to implementation
Madden 2022^16^	Pharmacists working in general practice within PCNs	Structured medication review (SMR) service within PCNs	Qualitative interview study	Themes related to early implementation of SMR service

CCGclinical commissioning groupPCNsprimary care networksRCTrandomised controlled trial

All the interventions involved medication review in some form. Distinctive features of interventions included use of IT to identify patients for review^13^,^1517 18^; a key role for PIPs in medication management in care homes^23 24^; and employment of pharmacists by groups of general practices (primary care networks, PCNs) to provide a holistic patient-centred service specified by NHS England.^16^ Intervention characteristics are considered in more detail below.

Study designs used included one individual randomised controlled tril (RCT)^19^ and two cluster RCTs (CHIPPS^20 23^ and PINCER^13^), although the primary publications of the latter two trials fell outside the time period covered by this review. Two studies used an interrupted time series (ITS) design^17 18^ and five used qualitative approaches.^14^,^1620 24^ One study was a mixed-methods process evaluation.^23^ The remaining studies were described as service evaluations or quality improvement reports with an uncontrolled before versus after design.^21 22 25 26^

Included studies reported a wide range of outcomes (table 2). For further analysis, see below under ‘effects of interventions’ and ‘Implementation/system issues, respectively. None of the studies reported details of participants other than age and sex, making it difficult to assess equity, diversity and inclusion across the evidence base.

#### Intervention characteristics

Table 3 in online supplemental file 2 summarises the characteristics of the included interventions using the TIDieR Lite checklist. The table includes limited data extracted from studies cited by included studies but not themselves included in the review.^27^,^29^

**Table 3:** Summary of studies reporting effects of interventions

Reference	Intervention	Setting	Study design and sample size	Outcome measure and effect size
Alves 2019^21^	Medication review	Care homes	Service evaluation10 405 patient reviews over 5 years	Interventions by pharmacist
Baqir 2017^22^	Medication review	Care homes	Retrospective evaluation of quality improvement project422 residents in 20 care homes	Number and type of medications stopped19.5% reduction in number of medicines being prescribed relative to baseline
Peek 2020^17^	Safety medication dashboard	General practice	Interrupted time series43 general practices covering 235 595 people in Salford, Greater Manchester	Potentially hazardous prescribing (composite of 10 indicators)Potentially hazardous prescribing reduced by 27.9% (95% CI 20.3% to 36.8%, p*<*0.001) at 24 weeks and by 40.7% (95% CI 29.1% to 54.2%, p*<*0.001) at 12 months
Rodgers 2022^18^	Pharmacist-led IT-assisted intervention (PINCER)	General practice	Multiple interrupted time series393 general practices covering approximately 3 million patients	Indicators of potentially hazardous prescribingThe PINCER intervention was associated with a decrease in the rate of hazardous prescribing of 16.7% (adjusted odds ratio (aOR) 0.83, 95% CI 0.80 to 0.86) at 6 months and 15.3% (aOR 0.85, 95% CI 0.80 to 0.90) at 12 months postintervention
Syafhan 2021^19^	Pharmacist-led medicines optimisation	General practice	Individual randomised controlled trial (RCT)356 patients at risk of medication-related problems (MRPs) from 8 GP practices	Medication-related problems (MRP); Medicines Appropriateness Index (MAI)Median number of MRPs per intervention patient at 6 months was reduced from 3 to 0.5 (p<0.001) in patients who received the full intervention schedule. MAI scores were reduced (medications more appropriate) for the intervention group, but not for control group.
Thayer 2021^25^	Review of medicines and lifestyle risk factors	Care homes for adults with intellectual disabilities (ID)	Service evaluation160 care home residents with ID	Pharmacist interventions/recommendations and acceptance by GPs and psychiatrists
Twigg 2015^26^	Community pharmacist consultation including medication review	Community pharmacies	Service evaluation620 patients (aged over 65 years and prescribed ≥4 medications	Number of recommendations; falls, medication adherence, quality of life and costs at 6 months

The pharmacists involved in delivering the interventions were variously described as PIPs^23^; trained pharmacists and pharmacy technicians^13 18^; primary care pharmacists^21^; clinical pharmacists working in general practice^15^,^17^; GP practice-based pharmacists working as part of a wider primary care team^19^; community and specialist mental health pharmacists^25^; and community pharmacists and pharmacy team members.^26^ One study simply referred to ‘pharmacists’.^15^

Four interventions were explicitly stated to require training of pharmacists to deliver them^13 19 23 26^; the extent of training was described for three of these.^19 23 26^ Training pharmacists to deliver the PINCER intervention was described in a separate paper.^13^ Interventions were delivered with other primary care team members depending on the setting of the study and in some cases with staff employed by clinical commissioning groups (CCGs). In particular, only the CHIPPS study involved pharmacists with the power to prescribe medication independently; in other studies recommendations were passed to the patient’s GP or another medically qualified professional for implementation. Shared decision-making with patients and/or families was specifically reported for three interventions.^16 19 22^

Reporting of interventions varied between studies. Most studies reported the process of medication review including patient selection for review and the review itself in more detail than resulting follow-up actions. Two qualitative studies reported limited details of the review process,^14 16^ although a service specification was available for the NHS England SMR investigated by Madden *et al*.^16^ For studies where the intervention was primarily directed at improving medication review processes using general practice data,^13^,^15^ it was unclear whether there was a standard process to discuss findings with the patient and make changes to their prescriptions. All studies reporting on effectiveness of medication reviews stated that the person undertaking the review had access to relevant patient records.^17^,^1921 22 25 26^

Intensity of interventions was also variably reported. In the CHIPPS study, PIPs committed a minimum of 16 hours/month to deliver care to approximately 20 care home residents.^27^ Madden *et al* reported that SMR appointments were recommended to allow at least 30 min for review and shared decision-making.^16^ The medicines optimisation intervention evaluated by Syafhan *et al* involved up to three meetings between patient and pharmacist^19^ while the FOMM study in community pharmacies estimated times of 25 min for initial consultation, 10 min for monthly review and 11 min for quarterly review.^26^ Other studies reported that time and level of support allocated to interventions varied between and within CCG areas depending on local resources and priorities.^18 21^ Another measure of intervention intensity was the number of recommended actions, averaging 3.3/resident in care home residents with ID.^25^

Most included studies reported on a single round of medication reviews with variable periods of follow-up. As noted above, some interventions required multiple interactions between pharmacists and patients.

#### Effects of interventions

Seven studies reported on effects of pharmacist-led interventions in some form (table 3): three in general practice,^17^,^19^ three in care homes^21 22 25^ (including one in a care home for people with ID^25^) and one in community pharmacies.^26^

The strongest evidence for the effectiveness of interventionscame from the studies in general practice. The ITS studies of Peek *et al*^17^ and Rodgers *et al*,^18^ which used indicators of inappropriate prescribing to identify patients for intervention, reported significant decreases in inappropriate prescribing at 6 and 12 months after intervention (table 3). Estimated reductions were larger in Peek *et al* (27.9% and 40.7%) compared with Rodgers *et al* (16.7% and 15.3%).^17 18^ The 95% CIs of the two studies at 12 months did not overlap, suggesting some uncertainty about the magnitude of the effect. The randomised trial by Syafhan *et al*^19^ preferentially recruited patients based on prescription of six or more medications and a history of recent unplanned hospital admission. The intervention was associated with a reduction in medication-related problems in those who completed the full programme (up to three appointments) and an improvement in Medicines Appropriateness Index (MAI) scores.

Of the three studies set in care homes, only Baqir *et al* reported a direct effect on prescribing associated with medication review, a 19.5% reduction in number of prescribed medicines.^22^ Alves *et al*^30^ reported on pharmacist interventions and potential financial savings over 5 years. In the 1-year reported in detail, 24.5% of interventions involved deprescribing. Potential drug cost savings were estimated at £812 441 annually, of which £431, 493 (55%) was attributed to deprescribing. The study of Thayer *et al*^25^ differed from the others in involving care home residents with IDs. There was a high level of polypharmacy at baseline and pharmacists made an average of 3.3 interventions/recommendations per resident, of which 12.8% involved deprescribing. A large majority of pharmacist recommendations were accepted by GPs/psychiatrists caring for the residents.

One study in a community pharmacy setting recruited patients aged 65 or older who were prescribed four or more medications.^26^ Of 620 patients recruited, 441 (71.1%) completed the 6-month study. Pharmacists made 142 recommendations related to 110 patients, largely dealing with potentially inappropriate prescribing of non-steroidal anti-inflammatory drugs (NSAIDs) and PPIs or duplication of therapy. The study also reported a significant decrease in falls and improvements in medication adherence and quality of life at follow-up.

The review included two publications from the CHIPPS Care Homes Independent Pharmacist Prescriber Study) trial^20 23^ but the paper reporting effectiveness and safety results from this cluster RCT^31^ was published too late for formal consideration for inclusion in our review. The primary outcome was rate of falls, with Drug Burden Index (DBI) being one of the secondary outcomes. Fall rate at 6 months did not differ significantly between intervention and control groups but DBI was lower in the intervention group (mean 0.66 vs 0.73; adjusted rate ratio 0.83, 95% CI 0.74 to 0.92).

#### Implementation/system issues

Seven studies provided quantitative and/or qualitative evidence on factors affecting implementation of pharmacist-led interventions, of which four were performed in general practice^13^,^16^ and three in care homes.^20 23 24^

The general practice studies focused on different parts of the implementation pathway. Two dealt with implementation of IT systems to support detection of potentially hazardous prescribing^14 15^; one was a process evaluation of the PINCER trial^13^; and one focused on implementation of SMRs as recommended by NHS England in routine practice.^16^ The studies of IT-supported interventions were broadly positive about the potential for implementation and sustainability, but the study of NHS England’s SMR programme concluded that its early implementation failed to deliver the planned holistic and patient-centred approach.

### Other evidence

#### Conference abstracts

We included 11 conference abstracts (table 4), of which 2 were earlier reports of studies subsequently published as full papers.^30 32^ All of the included abstracts focused on intervention effects on prescribing and related outcomes.

**Table 4:** Summary of selected grey literature case studies

Setting	Name of initiative	Key findings	Comments
Brighton and Hove CCG	An evaluation of a clinical pharmacist medication review service in primary care	A total of 1300 patients were referred into the service and reviewed between April 2017 and March 2018; 9% of patients were deprescribed high-risk medicines	The target patient cohort of frail or older persons prescribed polypharmacy was identified from searches within GP clinical systems and through referrals from clinical practitioners, voluntary and social care services
Swale CCG	Medicines Optimisation Review Programme	In 2018/19, pharmacists and pharmacy technicians reviewed 5281 patients and made 3859 interventions, 37% for adverse drug reactions (ADRs). Estimated in-year cost savings were £239 546	Targeted at ‘high-risk’ patientsKey feature is use of technicians for less complex cases
NE Hampshire and Farnham CCG	Care homes pharmacist	Pharmacist accompanying GPs visiting care homes carried out over 250 medication reviews and 800 interventions. Average number of medicines per resident fell from 9.4 to 7.6	Limited data reported
NE Hampshire and Farnham CCG	Polypharmacy prescribing comparators	Tool developed by Wessex AHSN was used to identify patients at risk of harm, resulting in significant reductions in percentage of patients aged over 75 prescribed 15 or more medications and percentage with an anticholinergic burden score of 6 or more	Limited data reported

Five abstracts reported research in general practice, of which three involved patients with polypharmacy identified from the overall practice population.^33^,^35^ As a group, these three abstracts provided weak evidence of associations between pharmacist-led medication reviews and changes in medication and cost savings together with high levels of patient satisfaction (table 5).

**Table 5:** Summary of studies published as conference abstracts

Reference	Population	Intervention	Study design	Outcome measures and key findings
Alves 2016^30^	Care home residents	Medication review by primary care pharmacists linked to GP practices	Service evaluation (retrospective analysis and interviews)	Interventions by pharmacist; barriers and facilitatorsA total of 2916 interventions were made in 1047 patients, of which deprescribing represented 22%
Bryant 2019^33^	Primary care patients taking ten or more medications	Polypharmacy clinics in GP surgeries	Service evaluation (retrospective data analysis)	Reductions in prescribing; cost savings; hospital admissions avoidedApril 2017 to March 2018, 370 patients reviewed and £50 766.63 saved; figures for April to December 2018 were 209 and £17 942, respectively
Chauhan 2022^37^	Patients recently discharged from hospital	Postdischarge medication review by clinical pharmacist linked to GP practice	Formative service evaluation (uncontrolled)	Medication changes following review16/35 patients had medications changed; 74% (25/34) of changes were medications stopped
Din 2020^34^	Patients referred by GPs	Polypharmacy review clinics led by pharmacist-independent prescriber with shared decision-making	Service evaluation (uncontrolled)	Changes to medication, feedback from patients and MDTPharmacist medication reviews were effective, with positive feedback received from patients and members of the MDT. Deprescribing and inhaler counselling were the most common interventions.
Din 2022^36^	Primary care patients living with frailty	Frailty review involving pharmacist as part of MDT	Comparative cohort	Changes in medication (including cholinergic burden), practice contacts and fallsIntervention group had a reduction in total number of medications when compared with non-intervention cohort. Anticholinergic burden scores were reduced by a mean of 26%
Doherty 2020a, 2020b^38 39^	Care home residents	Medicines Optimisation in Older People involving case management by pharmacists	Uncontrolled before/after	Inappropriate prescribing; unplanned hospital admissions; GP visits; clinical interventionsInappropriate prescribing was highly prevalent at baseline (84.1%) but improved significantly from baseline (M=14.87, SD=13.11) to post-intervention (M=0.70, SD=2.04, Z=25.97, p<0.001).
Donyai 2017^35^	Patients aged at least 75 years and prescribed 15 or more medication	Pharmacist-led polypharmacy review clinic in primary care	Survey	Patient satisfaction and related outcomesOf the 166 patients who returned a satisfaction questionnaire (40% response rate), 83% found the service helpful, 13% did not, 2% did not know and 2% did not respond
Kolovetsios 2018^41^	Care home residents needing palliative care	Structured medication reviews carried out in agreement with patient, nurse, family/carer and GP	Service evaluation	Changes to medication, estimated cost savingsFrom January 2017 to January 2018, 574 medication reviews took place, resulting in 1787 suggested medication changes. Approximately 76% of these changes were agreed and actioned by patients' GPs, with estimated savings of £169 986.96.
Swift 2018^40^	Care home residents	Care home team (pharmacists and pharmacy technicians) delivering medication reviews and supporting care home staff	Service evaluation	Prescribing quality indicators (including reduced inappropriate polypharmacy); CQC ratingsMedication reviews were completed for 749 care home residents between August 2014 and March 2017. Of the recommendations made to prescribers, 85% were accepted and resulted in a reduction in inappropriate polypharmacy
Syafhan 2019^32^	Patients in participating GP practices at risk of MRPs	Pharmacist-supplemented care focusing on medication optimisation	Individual randomised controlled trial (RCT)	Number of medication-related problems (MRPs) and medication inappropriatenessA total of 356 adult patients (175 control and 181 intervention) were recruited. Among 108 intervention patients who had three pharmacist face-to-face contacts, 346 MRPs were identified at baseline and 83 MRPs at 6 months. Median values were 3 MRPs at baseline and 1 at 6 months (p<0.001).

Two abstracts reported on selected general practice populations. The only comparative study in this group reported that patients living with frailty who were reviewed by a pharmacist as part of a multidisciplinary team review had a reduction in total medications compared with a control cohort.^36^ When patients recently discharged from hospital were reviewed by a pharmacist working in their general practice, 16 out of 35 had changes made to their medication, with 74% of changes involving deprescribing.^37^

Turning to studies performed in care homes, two abstracts by Doherty *et al*^38 39^ evaluated an intervention entitled Medicines Optimisation in Older People which involved case management by pharmacists. The authors reported that inappropriate prescribing (based on the MAI) was highly prevalent at baseline×84%) but declined significantly following the intervention. Swift reported that a team comprising pharmacists and pharmacy technicians who both performed medication reviews and supported care home staff significantly reduced inappropriate polypharmacy (measured by prescribing quality indicators) between 2024 and 2017.^40^ For care home residents receiving palliative care, SMRs involving shared decision-making were associated with high rates of changes to medication (1787 suggested changes from 574 reviews, 76% of which were implemented) and associated cost savings.^41^

#### Grey literature case studies

We included reports of four case studies reporting on local initiatives in three areas of England (see table 4). Details of all case studies may be found in Annex C of the NOR report.^1^ Case studies were submitted by NHS organisations (mainly CCGs) and included varying amounts of data on intervention characteristics, support for implementation and outcome measures. Three interventions were delivered in general practice and one in care homes. The initiative developed by Swale CCG was distinctive in using pharmacy technicians to review less complex cases, although the initiative was targeted at patients considered high risk for ADRs. Although not classified as research, such case studies can provide useful data on implementation of interventions and outcomes achieved in routine practice

### Study quality

Quality assessment results using the MMAT are presented in online supplemental file 3. The results should be read in conjunction with the study strengths and limitations (see table 2 in online supplemental file 1).

Five different checklists within the MMAT were used to assess the 14 studies. The sample included one RCT^19^; six studies were classified as quantitative non-randomised^17 18 21 22 25 26^; one as quantitative descriptive^13^; one as mixed methods^23^ and five as qualitative.^14^,^1620 24^ All studies passed the screening questions (are there clear research questions? and do the collected data allow to address the research questions?)

The RCT by Syafhan *et al* was described as a pragmatic trial and was at relatively high risk of bias for this type of design. The trial did not achieve the planned number of participants and there was a high rate of attrition (about 30%), meaning that many participants did not receive the full intervention or provide outcome data. The trial also suffered from unclear reporting: method of randomisation and whether outcome assessors were blinded was not reported, making it difficult to assess overall risk of bias.

The quantitative non-randomised studies comprised four observational studies at high risk of bias because of the absence of a control group^21 22 25 26^ and two large ITS studies.^17 18^ The MMAT tool identified some limitations of these studies, including some risk of confounding and incomplete outcome data in one study.^18^ However, these were large studies conducted in routine practice and providing evidence of a statistically significant effect at 12 months postintervention. The process evaluations of the CHIPPS^23^ and PINCER^13^ studies both scored highly on the MMAT assessment.

The qualitative studies were generally of good quality, with sufficient data presented in support of conclusions and appropriate use of frameworks and thematic analysis to organise presentation of the findings. The study by Alharthi *et al*^20^ was a secondary analysis of data collected for another purpose, making it unclear whether qualitative data collection methods were adequate.

Using the system applied by the authors in previous studies of complex health service interventions,^12^ the overall strength of evidence was classified as borderline ‘stronger’ (generally consistent findings in multiple studies with a comparator group) for general practice, ‘weaker’ (generally consistent findings in one study with a comparator group design and several non-comparator studies or multiple non-comparator studies) for care homes and ‘very limited’ (single study) for community pharmacies.

### Effectively implemented interventions

Three research studies met the criteria for ‘effectively implemented’ interventions: the closely related PINCER^18^ and SMASH^17^ interventions in general practice and the Somerset model of medication review in care homes.^21^ Further examples of effectively implemented medication review in care homes were identified among the included conference abstracts.^38^,^41^ Case studies from Brighton and Hove and Swale CCGs appeared to report effectively implemented interventions targeted at high-risk patients in general practice (table 4). An evaluation of the early implementation of SMRs in PCNs indicated that the service as provided did not match the vision of a patient-centred holistic review with an emphasis on shared decision-making.^16^

## Discussion

### Summary of findings

In spite of its broad inclusion criteria, this review identified a relatively small number of studies of pharmacist-led interventions in UK primary care (14 peer-reviewed journal articles, 11 conference abstracts and 4 case studies). Overall, the bulk of evidence came from the care home sector but most of the better quality evidence was derived from studies conducted in general practice. The majority (8/14) of peer-reviewed papers were published in 2020 or later, suggesting that this is a developing area of research and practice in the context of encouraging patients to consult pharmacists initially for minor conditions and to increase pharmacists’ prescribing rights. It was encouraging that we identified a number of effectively implemented interventions and initiatives in both care homes and general practice.

#### Outcomes of effective interventions

This systematic review suggests that pharmacist-led interventions may reduce overprescribing in primary care settings in the UK, although more controlled studies are needed. The evidence is strongest for interventions implemented in general practice, where we identified a small randomised trial^19^ as well as two large quasi-experimental studies (ITS)^17 18^ and various uncontrolled studies and service evaluations. Evidence from care home settings was of lower quality with the exception of the CHIPPS study involving PIPs working in care homes.^23^ We located only one uncontrolled study based in UK community pharmacies.^26^

Although the direction of reported effects was clear, the limited number of controlled studies combined with the wide range of outcomes reported makes it difficult to estimate the size of any effect. For example, the two ITS studies using similar interventions reported markedly different reductions in measures of inappropriate prescribing at 6 and 12 months after implementation of the intervention.^17 18^ Uncertainty about effect sizes is increased because many of the studies lacked a control group and the results could have been influenced by other interventions in the health and social care system, for example, the Enhanced Health in Care Homes programme implemented in England. While our review focused primarily on outcomes related to prescribing, data on cost savings were also widely reported but the evidence was generally of low quality. We also found limited evidence of a link between reductions in measures of overprescribing and clinical outcomes, mainly because of lack of reporting. The CHIPPS study found no significant difference in its primary outcome of fall rate, although there was a reduction in DBI (a secondary outcome) in the intervention group at 6 months.^31^

#### Characteristics of effective interventions

The TIDieR Lite checklist provided a suitable structure for describing intervention characteristics for evidence synthesis purposes and this discussion follows its structure. Lack of reporting (especially of intervention intensity/frequency) was a limiting factor, as was reporting of varying intervention information across multiple publications.

Medication reviews were undertaken by pharmacists acting independently or in conjunction with GPs or care home staff. In a study in care homes for people with IDs, psychiatrists were also involved in review where appropriate.^25^ Pharmacy technicians were also involved in the PINCER study and could potentially have a greater role in relatively straightforward medication reviews.^13 18^ The included studies reported a variety of models of employment of pharmacists, including direct employment by GP practices, CCG Medicines Optimisation Teams, PIPs and community pharmacists. PCNs support employment of pharmacists by general practices and are the route chosen by NHS England to implement its model of SMR.

A major difference between settings is the need to identify patients requiring medication review in general practice, whereas most care home residents take multiple medications and could be considered candidates for review as part of their routine healthcare. A key element of the PINCER^13 18^ and SMASH^17^ interventions is the use of information technology to search electronic patient records efficiently across large numbers of general practices. Effective interventions were also characterised by attention to training and tools to support and sustain change in practice, for example, an ‘audit and feedback’ dashboard.^17^

Training of pharmacists and other staff to deliver interventions was reported to varying degrees, reflecting in part the publication channel of the research. For example, in the CHIPPS study PIPS had comprised 2 days of face-to-face instruction plus time in practice to develop relationships with the GP and care home staff.^23^ Specification and provision of appropriate training will be important for future development of pharmacist-led interventions, as also highlighted by the evaluation of NHS England’s SMR programme.^16^

Intervention intensity is another important factor in developing and delivering interventions. For the CHIPPS study, participating PIPs committed a minimum of 16 hours/month to the service.^23^ In general practice settings, NHS England recommended allowing 30 min for an SMR to give time for shared decision-making; this was interpreted to include time for preparation and writing up.^16^ This level of time requirement was also reported in the one study from a community setting, which estimated pharmacist time at 25 min for an initial consultation.^26^

In terms of intensity more generally, resourcing of interventions was reported to vary between commissioning groups (CCGs) depending on staff availability and other priorities.^13 18 21^ General practices varied in their use of a medication safety dashboard.^28^ Frequency of intervention was rarely reported, reflecting the short time frame of most included studies but it seems possible that there could be an ongoing need for review as patients get older and/or their health state changes.

#### Quality and risk of bias

The MMAT provided a good alternative to the use of multiple tools to assess risk of bias across diverse study designs. The only randomised trial assessed was designed as a pragmatic trial^19^ and the assessment confirmed a relatively high risk of bias. Publications from the CHIPPS study were included but the trial per se was not assessed for risk of bias because of the publication date of the main study report. Similarly, the PINCER intervention was supported by a randomised trial published in 2012, before the cut-off date for our review^42^). Well-conducted studies included in the review included large ITS studies,^17 18^ process evaluations^13 14 23^ and qualitative studies.^15 16^ Service evaluations and other lower-quality evidence tended to support higher quality studies by highlighting implementation and results achieved in routine practice, although a causal relationship between intervention and outcome remains uncertain in studies without a parallel control group.

#### Implementation barriers and facilitators

Implementation of pharmacist-led interventions was strongly influenced by factors affecting relationships between pharmacists and other health and care professionals, especially GPs. Given that most pharmacists are not prescribers, their recommendations around (de)prescribing need to be seen as ‘legitimate’ by GPs who are generally responsible for acting on the recommendations. This is facilitated by continuity at the system level, including existing links between pharmacists and GPs^23^ and good access to data.^14^ Jeffries *et al* reported that pharmacists took the lead in developing relationships with GPs, enabling a ‘learning health system’.^14^ The benefits of continuity at the system level could help to explain why early implementation of the SMR programme through the relatively new medium of PCNs was reported to be less successful than initially hoped.^16^

Implementation in care homes may be more complex than in general practice because of differences in systems and ‘culture’ between health and social care.^24^ Patients and their families may be supportive of medication review or oppose it based on real or perceived benefits of medication.^20^

The main message regarding implementation of pharmacist-led interventions across all settings is the need for involvement of all relevant stakeholders, preferably before starting the process of implementation, to understand the context and anticipate possible barriers.^24^

#### Identification of effectively implemented interventions/initiatives

Our simple criteria for ‘effectively implemented’ interventions/initiatives identified a number of examples published as research papers, conference abstracts or case studies (see ‘Effectively implemented interventions’ above). Despite limitations as research, some of the abstracts and case studies provided valuable information about how commissioners and providers had supported interventions and their commitment to continue the programme.^38^,^41^ In other studies, despite promising results, it was unclear whether the intervention would be implemented more widely.^19^

### Relationship to previous research

To our knowledge, this is the first systematic review of pharmacist-led interventions and initiatives specifically in UK settings. A scoping literature search identified 20 systematic reviews published between 2014 and 2023. The most recent review covered pharmacist integration into general practice to optimise prescribing and outcomes for patients with polypharmacy.^43^ The review included 23 studies, of which just three were from the UK. The conclusion that pharmacist integration probably reduced PIP and number of medicines (moderate certainty evidence) was in line with the findings of the present review. A 2016 systematic review by Riordan *et al* focused on pharmacist-led interventions to optimise prescribing in older community-dwelling adults in primary care.^44^ The authors concluded that pharmacist-led interventions may improve appropriateness of prescribing but the quality of evidence was low. The review included randomised and quasi-randomised studies published before December 2015, giving it limited overlap with our review.

### Strengths and limitations

The UK focus is both a strength and limitation of this review. We included evidence often excluded from systematic reviews to get as full a picture as possible of how pharmacist-led interventions are implemented and sustained in practice as well as their characteristics and effectiveness. The dual focus reflects the fact that pharmacist-led medicines optimisation and deprescribing in primary care is both an area of active research and of implementation within the healthcare system. Nevertheless, some of the evidence is not of high quality and we have tried to be appropriately cautious in our conclusions and identified implications.

Our broad review questions and UK focus resulted in a heterogeneous group of included studies. Meta-analysis was not possible so we performed a narrative synthesis in line with appropriate guidelines.^10 11^ The review was undertaken by a small but experienced team with expertise in systematic review methods and prescribing.

### Implications for service delivery

Several studies indicate that barriers to successful service delivery often arise from ‘system’ issues and differences in ‘culture’.^16 24^ Commissioners and providers engaged in developing new pharmacist-led services should ensure equitable access to data and information to avoid perceptions of ‘ownership’ by certain groups at the expense of others.^15^ In care homes, where medication review is an important component of healthcare for residents,^21^ implementation requires health and social care professionals to work together and ‘understand each other’s systems’.^24^ The holistic patient-centred SMR envisaged by NHS England may require culture change/training to foster an emphasis on direct patient contact and shared decision-making. Removal of financial incentives for PCNs to carry out SMRs as reported recently (https://pharmaceutical-journal.com/article/news/nhs-england-removes-financial-incentives-for-structured-medication-reviews-in-2023-2024) may complicate delivery, although the service remains a contractual requirement.

Services have been delivered successfully through CCGs Medicines Optimisation Teams with suitable training.^13 18^ The review also found evidence that services provided by PIPs appear to be a valid alternative to approaches requiring action by GPs or other medical professionals.^23^

### Implications for research

A major priority for research is to further evaluate the effectiveness of medication review in community pharmacy settings and how pharmacies might be best supported to deliver the service. A related need is for research to better understand public perceptions of community pharmacies as a setting for medication review and their pros and cons compared with alternative settings such as GP surgeries. Research is needed to support the development of the PIP role and how PIPs might best be used in combination with GPs and other professionals to support optimal prescribing across the health and care system.

Shared decision-making is key to the success of pharmacist-led interventions. Research is needed to better understand patient and family attitudes to shared decision-making in the context of deprescribing and the barriers and facilitators operating in different settings and with different professionals.

The present review focused on outcomes related to prescribing and a review of effects on patient and health system outcomes would be a logical follow-up. Finally, further research is needed to understand the effects of implementing pharmacist-led medication review in general practice on health inequalities and how to reduce unwarranted variations in service delivery between different practices or regions.

### Conclusions

The evidence base for pharmacist-led interventions varies widely in terms of quality but studies have consistently reported improvements relative to a comparator group or baseline. The diversity of interventions and outcomes reported makes it difficult to generalise about effect sizes but given the reported extent of the problem, even small relative reductions could be beneficial for patients and the health and care system.

The existing evidence base requires cautious interpretation because of a shortage of controlled studies and this is particularly the case for studies in community pharmacy settings. Further rigorous evaluation of interventions, particularly those delivered in community pharmacies, is required. Although not a focus of this review, there appears to be a shortage of high-quality economic evidence to guide decision-making by service commissioners and providers.

The problems encountered in the early implementation of NHS England’s SMR programme^16^ suggest a need for further research on the implementation of pharmacist-led interventions. Implementation of this type of interventions requires the involvement of all relevant stakeholders, preferably before starting the process of implementation, to understand the context and anticipate possible barriers.

## supplementary material

10.1136/bmjopen-2023-081934 online supplemental file 1

10.1136/bmjopen-2023-081934 online supplemental file 2

10.1136/bmjopen-2023-081934 online supplemental file 3

## Data Availability

Data are available on reasonable request.
