# Supplementary material for: Pharmacist-led primary care interventions to promote medicines optimisation and reduce overprescribing: a systematic review of UK studies and initiatives
Source: BMJ Open. 2024 Aug 7;14(8):e081934. doi: 10.1136/bmjopen-2023-081934 (PMC11407218; doi:10.1136/bmjopen-2023-081934)
Supplement: online supplemental file 1 [file bmjopen-14-8-s001.pdf]

SEARCH STRATEGIES in full (for Appendix / supplementary material)

Ovid MEDLINE(R) ALL <1946 to February 06, 2023>

1 Inappropriate Prescribing/ 4485

2 ((hazardous* or excessive* or inappropriate* or unnecessar* or nonessential or non-essential or inessential) adj3 prescri*).mp. 8188

3 (overprescri* or over-prescri*).mp. 1975

4 Polypharmacy/ or (polypharmacy or poly-pharmacy).ti,ab. 12777

5 1 or 2 or 3 or 4 21236

6 exp Primary Health Care/ or (primary health care or primary healthcare or primary care).mp. 289526

7 general practice/ or family practice/ 78114

8 (GP or general practi* or family practice or family physician* or community pharmac* or dental or dentist* or optometr* or optician*).mp. 751694

9 6 or 7 or 8 997387

10 (deprescri* or de-prescri*).mp. 2577

11 (structured medication review or medication reconciliation or medicine* optimi#ation or shared decision making or personalised care).mp. 16563

12 ((intervention* or initiative* or campaign*) adj3 (pharmacist* or pharmacy technician*)).mp. 3182

13 10 or 11 or 12 21842

14 5 and 9 and 13 540

15 *Medication Errors/ and 9 and 13 232

16 5 and 9 and pc.fs. 835

17 14 or 15 or 16 1416

18 limit 17 to yr="2013 -Current" 1152

19 remove duplicates from 18 1145

Embase <1974 to 2023 Week 05>

1 Potentially inappropriate medication/ 2458

2 unnecessary prescribing/ [+NT] 51

3 ((hazardous* or excessive* or inappropriate* or unnecessar* or nonessential or non-essential or inessential) adj3 prescri*).mp. 11262

4 (overprescri* or over-prescri*).mp. 3064

5 Polypharmacy/ or inappropriate polypharmacy/ or (polypharmacy or poly-pharmacy).ti,ab. 26382

6 1 or 2 or 3 or 4 or 5 39236

7 exp Primary Health Care/ or primary medical care/ or (primary health care or primary healthcare or primary care).mp. 281889

8 general practice/ or family practice/ 83634

9 (GP or general practi* or family practice or family physician* or community pharmac* or dental or dentist* or optometr* or optician*).mp. 766031

10 7 or 8 or 9 974647

11 (deprescri* or de-prescri*).mp. 3587

12 (structured medication review or medication reconciliation or medicine* optimi#ation or shared decision making or personalised care).mp. 28235

13 ((intervention* or initiative* or campaign*) adj3 (pharmacist* or pharmacy technician*)).mp. 6755

14 11 or 12 or 13 37710

15 6 and 10 and 14 813

16 6 and 10 and pc.fs. 308

17 15 or 16 1089

18 limit 17 to yr="2013 -Current" 903

19 remove duplicates from 18 886

APA PsycInfo <1806 to January Week 5 2023>

1 ((hazardous* or excessive* or inappropriate* or unnecessar* or nonessential or non-essential or inessential) adj3 prescri*).mp. 788

2 (overprescri* or over-prescri*).mp. 329

3 (polypharmacy or poly-pharmacy).mp. 3128

4 1 or 2 or 3 4078

5 (primary health care or primary healthcare or primary care).mp. 44486

6 (GP or general practi* or family practice or family physician* or community pharmac* or dental or dentist* or optometr* or optician*).mp. 34287

7 5 or 6 71196

8 (deprescri* or de-prescri*).mp. 336

9 (structured medication review or medication reconciliation or medicine* optimi#ation or shared decision making or personalised care).mp. 3969

10 ((intervention* or initiative* or campaign*) adj3 (pharmacist* or pharmacy technician*)).mp. 242

11 8 or 9 or 10 4505

12 4 and 7 and 11 44

MY EBSCOhost

#	Query	Limiters/Expanders	Last Run Via	Results
S16	S6 AND S10 AND S14	Limiters - Published Date: 20130101-20231231 Expanders - Apply equivalent subjects Search modes - Boolean/Phrase	Interface - EBSCOhost Research Databases Search Screen - Advanced Search Database - CINAHL	307
S15	S6 AND S10 AND S14			327
S14	S11 OR S12 OR S13			13,914
S13	(intervention* or initiative* or campaign*) n3 pharmacist*			1,981
S12	"structured medication review" or "medication reconciliation" or "medicine* optimi#ation" or "shared decision making" or "personalised care" or "personalized care"			10,941
S11	deprescri* or de-prescri*			1,345
S10	S7 OR S8 OR S9			336,381
S9	("primary care" or "primary health care" or "primary healthcare" or "primary medical care") OR (GP or "general practi*" or "family practi*" or "family physician*" or "community pharmac*" or dental or dentist* or optometrist* or optician*)			333,015
S8	(MH "Family Practice")			26,910
S7	(MH "Primary Health Care") OR (MH "Physicians, Family")			90,488
S6	S1 OR S2 OR S3 OR S4 OR S5			12,727

S5	polypharmacy or poly-pharmacy	7,664
S4	(MH "Polypharmacy (Saba CCC)") OR (MH "Polypharmacy+")	5,635
S3	overprescri* or "over prescri*"	1,026
S2	(hazardous* or excessive* or inappropriate* or unnecessar* or nonessential or non-essential or inessential) n3 prescri*	4,996
S1	(MH "Inappropriate Prescribing")	3,448

Search Name: **THE COCHRANE LIBRARY**

Date Run: **08/02/2023 13:50:34**

Comment:

ID	Search	Hits
#1	MeSH descriptor: [Inappropriate Prescribing] explode all trees	234
#2	MeSH descriptor: [Polypharmacy] explode all trees	312
#3	((hazardous* or excessive* or inappropriate* or unnecessar* or nonessential or "non essential" or inessential) near/3 prescri*):ti,ab,kw	771
#4	(overprescri* or "over-prescri*"):ti,ab,kw	161
#5	(polypharmacy or poly-pharmacy):ti,ab,kw	1288
#6	#1 or #2 or #3 or #4 or #5	2045
#7	MeSH descriptor: [Primary Health Care] explode all trees	9989
#8	MeSH descriptor: [General Practice] explode all trees	2877
#9	MeSH descriptor: [Family Practice] explode all trees	2242
#10	("primary health care" or "primary healthcare" or "primary care"):ti,ab,kw	24053
#11	(GP or "general practi*" or "family practice" or "family physician*" or "community pharmac*" or dental or dentist* or optometr* or optician*):ti,ab,kw	44879
#12	#7 or #8 or #9 or #10 or #11	70362
#13	MeSH descriptor: [Deprescriptions] explode all trees	68
#14	(deprescri* or de-prescri*):ti,ab,kw	364
#15	("structured medication review" or "medication reconciliation" or "medicine* optimi*" or "shared decision making" or "personalised care" or "personalized care"):ti,ab,kw	2425
#16	((intervention* or initiative* or campaign*) near/3 (pharmacist* or pharmacy)):ti,ab,kw	1559
#17	#13 or #14 or #15 or #16	4166
#18	#6 and #12 and #17	130

(3 reviews, 127 trials)
