# Supplementary material for: Pharmacist-led primary care interventions to promote medicines optimisation and reduce overprescribing: a systematic review of UK studies and initiatives
Source: BMJ Open. 2024 Aug 7;14(8):e081934. doi: 10.1136/bmjopen-2023-081934 (PMC11407218; doi:10.1136/bmjopen-2023-081934)
Supplement: online supplemental file 2 [file bmjopen-14-8-s002.pdf]

## Data extraction tables

### Table 1: Study characteristics of included research studies (full data extraction table)

| Study ID | Study design/sample size | Setting | Intervention | Key findings | Authors' conclusions | Study strengths/limitations |
|---|---|---|---|---|---|---|
| Alharthi 2023[18] | Secondary analysis of qualitative interview data<br><br>11 pharmacist independent prescribers (PIPs) who participated in a cluster randomised trial | Care homes in England and Scotland | Integration of PIPs into care homes to improve medication management | Factors that acted as both enablers and barriers were PIP relationship with General Practitioner (GP), care home staff and residents/families, awareness of the PIP role and family trust in PIPs' deprescribing activities *(social influences)*; PIPs' independent prescribing confidence, previous experience and ability dealing with residents' medications *(beliefs about capabilities)*; understanding of PIP role and PIP confidence in their role as an independent prescriber *(social/professional role and identity)*; access to residents' records, deprescribing decision support, regular follow-up from care home staff, resident difficulties with medications, | PiPs' involvement in care homes is influenced by numerous barriers and enablers that can be addressed to improve intervention effectiveness | Strengths: Diverse PIP contexts and perspectives on deprescribing; theory-informed analysis using Theoretical Domains Framework to identify barriers and enablers<br><br>Limitations: Only PIP perspective considered; analysis used data from interviews focused on the whole intervention process rather than exclusively on deprescribing |

| | | | | | | |
|---|---|---|---|---|---|---|
| | | | | teamwork, and time restraints *(environmental context and resources)*. Belief that the negatives of deprescribing outweigh benefits regarding certain medications *(beliefs about consequences) acted as a barrier.* | | |
| Alves 2019[19] | Service evaluation<br><br>10,405 patient reviews over 5 years | Care homes in Somerset | Medication review by primary care pharmacists linked to GP practices | Pharmacists made 23,955 interventions (mean 2.3 per patient) from the 10,405 patient reviews undertaken. 16.1% of interventions were related to safety. Potential drug cost savings were estimated at £812,441 over 5 years, of which £431, 493 (53%) was attributed to deprescribing | Medication reviews undertaken by primary care pharmacists in care homes generate a wide range of interventions, commonly involving deprescribing. The service contributes to the continuous optimisation of prescribing and monitoring of medicines and offers potential drug cost savings. | Strengths: Collection of data from 'real world' implementation of intervention over 5 years<br><br>Limitations: No control group, cost saving estimates not based on full economic evaluation |
| Baqir 2017[20] | Retrospective evaluation of quality improvement project<br><br>422 residents in 20 care homes | Care homes in two CCG areas in North East England | Medicines optimisation by a pharmacist acting independently or jointly with a GP. Shared decision making with the patient or their advocate | Of the 422 patients reviewed, 298 (70.6%) had at least one medicine deprescribed with 704 medicines (19.5%) being stopped. There was no statistically significant difference between pharmacist only and pharmacist plus GP in terms of deprescribing. Assuming that each medicine stopped would have been taken for another year, annualised cost savings were estimated at £65,471 | Medicines optimisation reviews can lead to a reduction in polypharmacy for care home residents through a deprescribing process. Patients' medicine regimens were simplified and optimised while making financial savings for the NHS | Strengths: Compares two approaches to delivering medication review<br><br>Limitations: Short-term uncontrolled study; intervention quality/fidelity not measured |

| | | | | | | |
|---|---|---|---|---|---|---|
| Birt 2021[21] | Mixed methods process evaluation of cluster RCT<br><br>Intervention arm comprised 25 triads: Care homes (staff and up to 24 residents), GP and pharmacist Independent Prescriber (PIP); 22 PIPs contributed data | Care homes in England, Scotland and Northern Ireland | Integration of PIPs into care homes to assume central responsibility for medicines management | All stakeholders reported some benefits from PIPs having responsibility for medicine management and identified no safety concerns. PIPs reported an increase in their knowledge and identified the value of having time to engage with care home staff and residents during reviews. PIPs recorded 566 clinical interventions, many involving deprescribing; 93.8% of changes were sustained at 6 months. For 284 (50.2%) residents a medicine was stopped, and for a quarter of residents, changes involved a medicine linked to increased falls risk. Qualitative data indicated participants noted increased medication safety and improved resident quality of life. Contextual barriers to implementation were apparent in the few triads where PIP was not known to the GP and care home before the trial. In three triads, PIPs did not deliver the intervention. | The intervention was generally implemented as intended, and well-received by most stakeholders.<br>Whilst there was widespread deprescribing, contextual factors effected PIP engagement. Implementation was most effective when communication pathways between PIP and GP had been<br>previously established. | Strengths: Involved three UK nations with differing healthcare systems; used study records to supplement qualitative data<br><br>Limitations: Interview participants may not be representative; limited access to care home residents |
| Howard 2014[11] | Process evaluation of data from cluster RCT | General practice surgeries in an 80 km | Pharmacist-led IT enabled intervention (PINCER). | Pharmacists judged 72% (95% CI 70, 74; 1463/2026) of cases of hazardous medicines management to be clinically | Recommendations from the pharmacists were broadly | Strengths: Uses data from a large cluster RCT |

| | | | | | | |
|---|---|---|---|---|---|---|
| | 36 intervention and 36 control practices; 1946 patients identified as at risk in intervention practices | radius around Manchester and Nottingham | Patients potentially at risk from hazardous medicines management were identified using Quest Browser software to search GP electronic records. Intervention practices were assigned a pharmacist who educated practice staff about medication management and recommended improvements to practice. Pharmacists also reviewed cases of potentially hazardous medication | relevant. Pharmacists recommended 2105 interventions in 74% (95% CI 73, 76; 1516/2038) of cases and 1685 actions were taken in 61% (95% CI 59, 63; 1246/2038) of cases; 66% (95% CI 64, 68; 1383/2105) of interventions recommended by pharmacists were completed and 5% were accepted by GPs but not completed at the end of the pharmacists' placement; the remaining recommendations were rejected or considered not relevant by GPs. | acceptable to GPs and led to ameliorative action in the majority of cases. It seems likely that the approach used by the PINCER pharmacists could be employed by other practice pharmacists following appropriate training. | Limitations: Pharmacists did not record detailed reasons for their judgements and these were not peer reviewed |

| | | | | | | |
|---|---|---|---|---|---|---|
| | | | and recommended interventions to GPs | | | |
| Jeffries 2017[13] | Qualitative realist evaluation<br><br>Interviews: 3 GPs, 2 CCG pharmacists; Focus groups: 2 GPs, 4 community pharmacists, 4 patients, 4 practice managers | CCG in the South of England | Electronic Medicines Optimisation System (EMOS). The EMOS is intended to facilitate clinical audits of prescribing activity to identify patients at risk of adverse drug events (ADEs) | Effective use of the EMOS depended upon engagement with the system, the flow of information between different health professionals centrally placed at the CCG and those locally placed at individual general practices, and upon adaptation of work practices to facilitate the use of the system. The use of the system was undermined by perceptions of ownership, lack of access, lack of knowledge and awareness, and time pressures. | The use of an electronic medicines optimisation system may improve medication safety in primary care settings by identifying those patients at risk of an ADE. To fully realise the potential benefits there needs to be better utilisation across primary care and with a wider range of stakeholders. Engaging with all potential stakeholders and users prior to implementation might allay perceptions that the system is owned centrally and increase knowledge of the potential benefits. | Strengths: Realist methodology enabled detailed examination of how the EMOS was used and its potential effects<br><br>Limitations: Study involved only one CCG so may not be representative |
| Jeffries 2018[12] | Qualitative process evaluation<br><br>28 staff members from 23 general practices (9 GPs, 12 pharmacists, 7 other GP staff) | 43 general practices in Salford, Greater Manchester | Electronic audit and feedback surveillance dashboard to identify patients potentially at risk of hazardous prescribing or | Engagement with the dashboard involved a process of ‘sense-making’ by pharmacists. The intervention helped to build respect, improve trust and develop relationships between pharmacists and GPs. Collaboration and communication between pharmacists and clinicians was primarily initiated by | Medicine optimisation in primary care may be enhanced by the implementation of a pharmacist-led electronic audit and feedback system. This intervention established a rapid learning health system that enabled data from electronic health records to be used to make changes in practice to improve patient care. | Strengths: Use of Normalization Process Theory as a framework to understand implementation<br><br>Limitations: Evaluation team also developed the intervention; number of follow-up |

| | | | | | | |
|---|---|---|---|---|---|---|
| | | | monitoring of medicines | pharmacists and was important for establishing the intervention. | | interviews was limited |
| Lane 2020[22] | Qualitative focus groups and interviews<br><br>85 (72 in focus groups and 13 in semi-structured interviews) | Care homes (4 sites in England (2), Scotland and Northern Ireland) | Integration of PIPs into care homes to take responsibility for medicines management | A PIP service was seen as offering benefits for residents, care homes and doctors but stakeholders raised challenges including agreement on areas where PIPs might prescribe, contextual barriers in chronic disease management, PIPs' knowledge of older people's medicine, and implementation barriers in integrated team-working and ensuring role clarity. Introducing a PIP was welcomed in principle but conditional on: a clearly defined PIP role communicated to stakeholders; collaboration between doctors, PIPs and care-home staff; and dialogue about developing the service with residents and relatives. | The overarching theme from this research was that everyone must "understand each other's systems". In particular, PIPs need to understand care homes' systems in advance of implementing a new service | Strengths: Purposively selected sample; use of TDF as a framework to analyse data<br><br>Limitations: Data relate to proposed service model in advance of implementation |
| Madden 2022[14] | Qualitative interview study<br><br>10 newly appointed pharmacists working in primary care | General practice in England | Structured medication review (SMR) for people at risk of harm or medication-related problems | SMR implementation was largely delegated to individual pharmacists. Established pharmacists appeared more ready for implementation than newly appointed staff. New pharmacists were learning about working in primary care settings and tended to follow procedures | Early implementation of SMRs did not match the intention of providing patients with a holistic review and shared decision-making. The authors identified an important opportunity cost of SMR implementation without prior adequate skills | Strengths: based on detailed, in-depth interviews<br><br>Limitations: Authors note interviews need to be complemented by data on actual |

| | | | | | | |
|---|---|---|---|---|---|---|
| | networks (PCNs) in Northern England; 10 established pharmacists working in GP practices in other PCNs | | | with which they were already familiar, particularly when they lacked patient-facing expertise. Implementation was affected by ongoing backlogs and workforce issues in general practices | development, testing, and refining | practice and longer term follow-up |
| Peek 2020[15] | Interrupted time series<br><br>43 general practices covering 235,595 people in Salford, Greater Manchester | General practice in England | Pharmacist-led Safety Medication dASHboard (SMASH). SMASH involved (1) training of clinical pharmacists to deliver the intervention; (2) a web-based dashboard providing actionable, patient-level feedback; and (3) pharmacists reviewing individual at-risk patients, | The study used an interrupted time series analysis of rates (prevalence) of potentially hazardous prescribing and inadequate blood-test monitoring, comparing observed rates post-intervention to extrapolations from a 24-month pre-intervention trend. At baseline, 95% of practices had rates of potentially hazardous prescribing (composite of 10 indicators) between 0.88% and 6.19%. The prevalence of potentially hazardous prescribing reduced by 27.9% (95% CI 20.3% to 36.8%, $p <$ 0.001) at 24 weeks and by 40.7% (95% CI 29.1% to 54.2%, $p <$ 0.001) at 12 months after introduction of SMASH. The rate of inadequate blood-test monitoring (composite of 2 indicators) reduced by 22.0% | The SMASH intervention was associated with reduced rates of potentially hazardous prescribing and inadequate blood-test monitoring in general practices. This reduction was sustained over 12 months for prescribing but not for monitoring of medication. There was a marked reduction in the variation in rates of hazardous prescribing between practices. | Strengths: Authors noted pragmatic design, evaluation of clinically relevant outcomes and large number of practices taking part<br><br>Limitations: Not a randomised study so possibility of unrecognised confounding cannot be excluded |

| | | | | | | |
|---|---|---|---|---|---|---|
| | | | and initiating remedial actions or advising GPs on doing so. | (95% CI 0.2% to 50.7%, *p* = 0.046) at 24 weeks; the change at 12 months (23.5%) was no longer significant (95% CI −4.5% to 61.6%, *p* = 0.127). After 12 months, 95% of practices had rates of potentially hazardous prescribing between 0.74% and 3.02%. | | |
| Rodgers 2022[16] | Multiple interrupted time series<br><br>393 general practices covering approximately 3 million patients | General practice in the East Midlands region of England | Pharmacist-led IT intervention to reduce hazardous prescribing (PINCER) | Successive groups of general practices received the PINCER intervention between September 2015 and April 2017. Eleven prescribing safety indicators were used to identify potentially hazardous prescribing and data were collected over a maximum of 16 quarterly time periods.<br>PINCER was implemented in 370 (94.1%) of 393 general practices; data were successfully extracted from 343 (92.7%) of these practices. For the primary composite outcome, the PINCER intervention was associated with a decrease in the rate of hazardous prescribing of 16.7% (adjusted odds ratio (aOR) 0.83, 95% confidence interval (CI) 0.80 to 0.86) at 6 | The PINCER intervention, when rolled out at scale in routine clinical practice, was associated with a reduction in hazardous prescribing by 17% and 15% at 6 and 12 months post-intervention. The greatest reductions in hazardous prescribing were for indicators associated with risk of GI bleeding. These findings support the wider national rollout of PINCER in England. | Strengths: Suggests intervention was implemented successfully in routine practice and was associated with significant reductions in hazardous prescribing<br><br>Limitations: The authors adjusted for calendar time and practice, but since this was an observational study, the findings may have been influenced by unknown confounding factors or behavioural changes unrelated to |

| | | | | | | |
|---|---|---|---|---|---|---|
| | | | | months and 15.3% (aOR 0.85, 95% CI 0.80 to 0.90) at 12 months post-intervention. The unadjusted rate of hazardous prescribing reduced from 26.4% to 20.1% at 6 months and 19.1% at 12 months. The greatest reduction was for hazardous prescribing indicators related to GI bleeding | | the PINCER intervention. Data were also not collected for all practices at 6 and 12 months post-intervention |
| Syafhan 2021[17] | Individual RCT<br><br>356 patients at risk of medication-related problems (MRPs) from 8 GP practices | General practice in England (6 practices) and Northern Ireland (2) | Medicines optimisation with shared decision-making and agreed treatment goals. Intervention repeated at 2 and 4 months, building on progress towards agreed goals | Median number of MRPs per intervention patient at 6 months was reduced from 3 to 0.5 ($p < 0.001$) in patients who received the full intervention schedule. Medication Appropriateness Index (MAI) scores were reduced (medications more appropriate) for the intervention group, but not for control group patients.<br>Using the intention-to-treat (ITT) approach, the number of telephone consultations in intervention group patients was reduced and different from the control group. No significant differences between groups were found in unplanned hospital admissions, length of hospital stay, number of A&E attendances or outpatient visits. The mean overall healthcare | The pharmacist service reduced MRPs, inappropriateness of medications and telephone consultations in general practice in a cost-effective manner | Strengths: Pragmatic randomised design<br><br>Limitations: Sample smaller than planned; high loss to follow-up; MRP analysis only covered patients who attended 3 appointments |

| | | | | | | |
|---|---|---|---|---|---|---|
| | | | | cost per intervention patient fell from £1041.7 ± 1446.7 to £859.1 ± 1235.2 ($p$ = 0.032). Cost utility analysis showed an incremental cost per patient of − £229.0 (95% CI − 594.6, 128.2) and a mean QALY gained of 0.024 (95% CI − 0.021 to 0.065),. indicative of a health status gain at a reduced cost (2016/2017). | | |
| Thayer 2021[23] | Service evaluation<br><br>160 care home residents with intellectual disabilities (ID) | Care homes for people with ID in the Wirral | Pharmacist review of residents' medicines and lifestyle risk factors between November 2019 and May 2020. | The 160 residents were prescribed 1207 medicines, 74% were prescribed ⩾5 medicines and 507 interventions/recommendations were made, averaging 3.3 per resident. The highest proportion (30.4%) were lifestyle risk related, while changing and stopping medicines accounted for 17.9% and 12.8%, respectively. Of the recommendations discussed with GPs/psychiatrists, 86% were accepted. | There was considerable polypharmacy among the residents and a high level of pharmacists' interventions/recommendations about medicines and lifestyle risk, most of which were accepted by GPs/psychiatrists. Wider adoption of collaborative pharmacist review models could have benefits for residential populations with ID and potentially reduce pressure on other health services | Strengths: Drew on skills of pharmacists from different sectors to address wide range of care needs; recommendations addressed national priorities<br><br>Limitations: Study limited to one CCG area; limited access to patient records; observational study with no control/comparator arm |
| Twigg 2015[24] | Service evaluation | Community pharmacies in England | Four or More Medicines (FOMM) support | Of 620 patients recruited, 441 (71.1%) completed the 6-month study period. Pharmacists made 142 | By focussing on patients over the age of 65 years with four or more medicines, community pharmacists can improve | Strengths: Large sample of patients and providers; use of |

| | | | | | | |
|---|---|---|---|---|---|---|
| | 620 patients (aged over 65 years and prescribed ≥ 4 medications) | | service. Patients were invited to participate in the service by the community pharmacy team. The pharmacist held regular consultations with the patient and discussed risk of falls, pain management, adherence and general health. They also reviewed the patient's medication using STOPP/START criteria. Data were analysed for the first 6 months of participation in the service. | recommendations to prescribers in 110 patients, largely centred on potentially inappropriate prescribing of NSAIDs, PPIs or duplication of therapy. At follow-up, there was a significant decrease in the total number of falls experienced and a significant increase in medicine adherence and quality of life. Cost per quality-adjusted life year estimates ranged from£11 885 to £32 466 depending on the assumptions made. | medicine adherence and patient quality of life. | validated outcome measures<br><br>Limitations: No control/comparator group; authors note some patients were probably reviewed independently by their GP during the study period; relatively high attrition rate |

Appendix Table 2: TIDieR Lite for UK pharmacist studies

| **Intervention name and study ID(s)** | **By whom** | **What** | **Where** | **Intensity** | **How often** |
|---|---|---|---|---|---|
| CHIPPS<br><br>Alharthi 2023[18]; Birt 2021[21]; Lane 2020 [22]; Bond 2020[25]; Holland 2023[29] | Trained pharmacist independent prescribers (PIPs). The training programme comprised 2 days of face-to-face instruction, time in practice to develop relationships with the GP and care home staff, and to address any self-assessed competency gaps supported by a mentor, and a formal final sign-off by a GP independent of the research | PIP, in collaboration with the care home resident's GP, assumes responsibility for managing the medicines of the resident, including:<br>• Reviewing resident's medication and developing and implementing a pharmaceutical care plan<br>• Assuming prescribing responsibilities<br>• Supporting systematic ordering, prescribing and administration processes with each care home, GP practice and supplying pharmacy where needed<br>• Providing training in care home and GP practice<br>• Communicating with GP practice, care home, | Participating care homes | PIPs committed a minimum of 16 hours/month to deliver the service. Each PIP provided care to approximately 20 residents | PIPs visited care homes weekly over 6 months |

| | | | | | |
|---|---|---|---|---|---|
| | | supplying community pharmacy and study team | | | |
| Care home medication reviews<br><br>Alves 2019[19] | Primary care pharmacists and GPs in Somerset CCG area and CCG staff | Medicines optimisation visits to care homes. Primary care pharmacists visited homes on behalf of GP practices; GPs could participate in visits or hold discussions with pharmacists prior to the visit; screening of safety interventions was done by CCG pharmacist leads | Care homes with and without nursing in Somerset | The time and level of support allocated for the service was agreed with the respective CCG Locality Pharmacist Manager and influenced by a number of factors such as engagement from GP practices; primary care pharmacists' availability; skills and confidence; number of care home patients registered with each GP practice; and geographic area covered by the prescribing support pharmacists | The aim of the programme was to offer at least one visit to as many care homes as possible (appears to be one visit per year but not explicitly stated) |
| Shine Medication Optimisation Project<br><br>Baqir 2017[20] | Pharmacists together with care home nurses and other members of the multi-disciplinary team (MDT), including GPs and mental health professionals as needed. Two different models: pharmacists made prescribing decisions (as part of shared decision-making) independently or in conjunction with GPs | A notes based, pharmacist-led review of medicines, where the Northumbria 3Q approach was applied to each medicine, that is, was there an indication, was the indication appropriate and was it safe?. Additionally, medicines missing that could be beneficial (eg, START medicines) were identified. This was followed by a MDT meeting where the information from the pharmacist-led review was discussed and an action plan was formulated. Whenever possible, the final decisions were made with patients and their families. After the review, | Care homes in North East England | Intensity of intervention not reported. Prescribing decisions could be made by pharmacists alone or in conjunction with GPs | Once, as a funded quality improvement (QI) project |

| | | | | | |
|---|---|---|---|---|---|
| | | the project database was updated to show medicines taken before review, medicines stopped, started or changed and any other interventions made. | | | |
| PINCER<br><br>Howard 2014[11]; Rodgers 2022[16] | Pharmacists specifically trained to deliver the intervention; GPs, other practice staff and pharmacy technicians involved in implementation | Computer systems of general practices are searched to identify patients at risk of potentially hazardous prescribing using a set of prescribing safety indicators. Pharmacists then provide an educational outreach intervention where they meet with GPs and other practice staff to:<br>• Discuss the search results and highlight the importance of the hazardous prescribing identified using brief educational materials. These feedback sessions were to be held straight after running the searches and then at regular intervals.<br>• Agree on an action plan, retained within the practice, for | General practices | When PINCER was rolled out in the East Midlands, time spent by pharmacists delivering the intervention varied by CCG depending on the resourcing level of the local Medicines Optimisation Team | Data collected quarterly up to 12 months after starting the intervention[16] |

| | | | | | |
|---|---|---|---|---|---|
| | | reviewing patients identified as high risk and improving prescribing and medication monitoring systems using root cause analysis<br>Pharmacists (sometimes supported by pharmacy technicians) then work with, and support, general practice staff to implement the agreed action plan, sometimes making the<br>necessary changes themselves | | | |
| Eclipse Live (electronic medicines optimisation system (EMOS))<br><br>Jeffries 2017[13] | Developed by a private company (Eclipse Solutions) and made available to stakeholders (including doctors, pharmacists, practice managers and patients) by a CCG in the South of England | Web-based user interface which securely extracts patient data from general practice patient records. Accessed separately from the GPs' clinical systems, it allows different stakeholders access to real time anonymized<br>patient data including medical histories of diagnoses, prescribed medications and test results. The EMOS is intended to facilitate clinical audits of prescribing activity to identify patients at risk of ADEs, or not appropriately monitored. | General practices covered by the participating CCG | Not reported (qualitative study) | Not reported (qualitative study) |

| | | | | | |
|---|---|---|---|---|---|
| | | Patients can access the system through a “Patient Passport” | | | |
| Safety Medication dASHboard (SMASH)<br><br>Jeffries 2018[12]; Peek 2020[15]; Jeffries 2020[26] | Clinical pharmacists working in general practices and other general practice staff | Pharmacists were trained to deliver the intervention and apply root cause analysis techniques to identify, explore, resolve, and prevent medication errors in partnership with general practice staff. Pharmacists and practice staff were given access to a web-based, interactive dashboard that provided feedback on 12 indicators of potentially hazardous prescribing. The dashboard also provided practice-level summary data as well as educational material. | General practices covered by the participating CCG | Practices interacted with the dashboard a median of 12.0 (interquartile range, 5.0–15.2) times per month during the first quarter of use. Over time, dashboard use transitioned towards regular but less frequent (median of 5.5 [3.5–7.9] times per month) checks to identify and resolve new cases. The frequency of dashboard use was higher in practices with a larger number of at-risk patients. | Dashboard was updated daily. Frequency of use varied by practice and over time (see previous column) |
| Structured Medication Review (SMR)<br><br>Madden 2022[14]; Stewart 2021[27] | Clinical pharmacists within general practice primary care networks (PCNs) | Invited, personalised, holistic review of all medicines and their benefits to health for people at risk of harm or medicine-related problems | General practices | Reviews are recommended to be scheduled for at least 30 minutes to allow time for shared decision-making | Once |
| Medicines optimisation intervention | GP practice-based pharmacists operating as part of the wider primary care team | Each pharmacist received 2 days of intensive specialist training | Eight general practices in four regions of the UK | Initial meeting with further appointments available at 2 and 4 months building on patient progress towards agreed goals | Once per patient (up to three appointments) |

| | | | | | |
|---|---|---|---|---|---|
| Syafhan 2021[17] | | on medicines optimisation (including training on motivational interviewing). The intervention included: review of patient records prior to meeting; medication history; individual medicines optimisation plan that could include recommending/making changes to medication regimens (in collaboration with GPs), personalised education and counselling on medication management, the correct use of medication administration devices and lifestyle factors; and an agreed list of treatment goals. Pharmacists could also refer patients to another health professional within the practice.<br>Having completed the intervention, the pharmacist produced a short report for the patient's GP outlining actions taken and any further recommendations requiring GP input | | | |

| | | | | | |
|---|---|---|---|---|---|
| Collaborative pharmacist review<br><br>Thayer 2021[23] | Community and specialist mental health pharmacists | Medicine review using a structured framework based on recommendations of the 2018 Learning Disability Mortality Review (LeDeR) report. Pharmacists visited care homes to conduct the reviews using individual residents' care home records. The specialist mental health pharmacist also had access to the care record held by the Specialist Mental Health Trust, if the resident was under the Trust's care, and remote access to the local data sharing platform. Assessments included medicines adherence and burden (particularly the anticholinergic burden), respiratory care, vaccination status, constipation risk, sepsis prevention, dysphagia risk and lifestyle risk issues, especially smoking. Finally, pharmacists were asked to detail actions taken/advice provided, any recommendations made and make referrals, as necessary. Following the review, GP surgeries and psychiatrists were contacted by the pharmacists to arrange a review of their | Care homes for people with intellectual disabilities | 507 interventions/recommendations for 160 residents reviewed (3.3 per resident) | Once |

| | | | | | |
|---|---|---|---|---|---|
| | | recommendations. As the pharmacists were not prescribers, decisions on accepting recommendations were made by the resident's GP/psychiatrist (after reviewing the resident's full clinical record) in consultation with the pharmacists | | | |
| Four or More Medicines (FOMM) support service<br><br>Twigg 2015[24] | Community pharmacists and pharmacy team members | Pharmacists were trained via distance learning and face to face, which included how to use the various different tools and assessments. Training was then cascaded to other pharmacy members.<br>Patients were invited to participate in the service by the community pharmacy team. The pharmacist held regular consultations with the patient and discussed risk of falls, pain management, adherence and general health. They also reviewed the patient's medication using STOPP/START criteria. | Participating community pharmacies | Pharmacist time estimated at 25 minutes for initial consultation, 10 minutes for monthly review and 11 minutes for quarterly review | After the first consultation, patients met with the pharmacist on a regular basis depending on when they collected their repeat medication or they felt a need. |
