# Supplementary material for: Pharmacist-led primary care interventions to promote medicines optimisation and reduce overprescribing: a systematic review of UK studies and initiatives
Source: BMJ Open. 2024 Aug 7;14(8):e081934. doi: 10.1136/bmjopen-2023-081934 (PMC11407218; doi:10.1136/bmjopen-2023-081934)
Supplement: online supplemental file 3 [file bmjopen-14-8-s003.pdf]

MMAT quality assessment results

Reference	Screening questions	Type of study	MMAT questions and answers
Alharthi 2023[18]	**S1. Are there clear research questions?** *Yes*<br><br>**S2. Do the collected data allow to address the research questions?** *Yes*	Qualitative	**1.1. Is the qualitative approach appropriate to answer the research question?** *Yes (identifying perceived barriers and facilitators)*<br>**1.2. Are the qualitative data collection methods adequate to address the research question?** *Can't tell (secondary analysis of existing data)*<br>**1.3. Are the findings adequately derived from the data?** *Yes*<br>**1.4. Is the interpretation of results sufficiently substantiated by data?** *Yes*<br>**1.5. Is there coherence between qualitative data sources, collection, analysis and interpretation?** *Yes (supported by use of Theoretical Domains Framework)*
Alves 2019[19]	**S1. Are there clear research questions?** *Yes*<br><br>**S2. Do the collected data allow to address the research questions?** *Yes*	Quantitative non-randomised	**3.1. Are the participants representative of the target population?** *Yes (care home residents)*<br>**3.2. Are measurements appropriate regarding both the outcome and intervention (or exposure)?** *Yes*<br>**3.3. Are there complete outcome data?** *Can't tell (partial data presented*)<br>**3.4. Are the confounders accounted for in the design and analysis?** *No (uncontrolled before/after study)*<br>**3.5. During the study period, is the intervention administered (or exposure occurred) as intended?** *Can't tell (fidelity not monitored)*
Baqir 2017[20]	**S1. Are there clear research questions?** *Yes*<br><br>**S2. Do the collected data allow to address the research questions?** *Yes*	Quantitative non-randomised	**3.1. Are the participants representative of the target population?** *Yes (care home residents)*<br>**3.2. Are measurements appropriate regarding both the outcome and intervention (or exposure)?** *Yes*<br>**3.3. Are there complete outcome data?** *Yes (all specified outcomes reported)*<br>**3.4. Are the confounders accounted for in the design and analysis?** *No (uncontrolled before/after study)*<br>**3.5. During the study period, is the intervention administered (or exposure occurred) as intended?** *Can't tell (interventions not externally validated)*

Birt 2021[21]	**S1. Are there clear research questions?** *Yes*<br><br>**S2. Do the collected data allow to address the research questions?** *Yes*	Mixed methods	**5.1. Is there an adequate rationale for using a mixed methods design to address the research question?** *Yes (qualitative and quantitative data relevant to process evaluation)*<br>**5.2. Are the different components of the study effectively integrated to answer the research question**? *Yes (integrated in results and discussion)*<br>**5.3. Are the outputs of the integration of qualitative and quantitative components adequately interpreted?** *Yes (see discussion)*<br>**5.4. Are divergences and inconsistencies between quantitative and qualitative results adequately addressed?** *Yes (page 11 column 2)*<br>**5.5. Do the different components of the study adhere to the quality criteria of each tradition of the methods involved?** *Yes*
Howard 2014[11]	**S1. Are there clear research questions?** *Yes*<br><br>**S2. Do the collected data allow to address the research questions?** *Yes*	Quantitative descriptive	**4.1. Is the sampling strategy relevant to address the research question?** *Yes*<br>**4.2. Is the sample representative of the target population?** *Yes (all interventions recorded)*<br>**4.3. Are the measurements appropriate?** *Yes*<br>**4.4. Is the risk of nonresponse bias low?** *Yes (data from intervention arm only)*<br>**4.5. Is the statistical analysis appropriate to answer the research question?** *Yes*
Jeffries 2017[13]	**S1. Are there clear research questions?** *Yes*<br><br>**S2. Do the collected data allow to address the research questions?** *Yes*	Qualitative	**1.1. Is the qualitative approach appropriate to answer the research question?** *Yes (explored factors perceived to affect adoption and implementation)*<br>**1.2. Are the qualitative data collection methods adequate to address the research question?** *Yes (interviews and focus groups)*<br>**1.3. Are the findings adequately derived from the data?** *Yes (context-mechanism-outcome groups identified)*<br>**1.4. Is the interpretation of results sufficiently substantiated by data?** *Yes*<br>**1.5. Is there coherence between qualitative data sources, collection, analysis and interpretation?** *Yes (supported by use of realist analysis)*
Jeffries 2018[12]	**S1. Are there clear research questions?** *Yes*	Qualitative	**1.1. Is the qualitative approach appropriate to answer the research question?** *Yes (explored factors perceived to affect adoption and implementation)*

	**S2. Do the collected data allow to address the research questions?** *Yes*		**1.2. Are the qualitative data collection methods adequate to address the research question?** *Yes (interviews)*<br>**1.3. Are the findings adequately derived from the data?** *Yes*<br>**1.4. Is the interpretation of results sufficiently substantiated by data?** *Yes (supported by relevant quotes)*<br>**1.5. Is there coherence between qualitative data sources, collection, analysis and interpretation?** *Yes (supported by use of Normalisation Process Theory)*
Lane 2020[22]	**S1. Are there clear research questions?** *Yes*<br><br>**S2. Do the collected data allow to address the research questions?** *Yes*	Qualitative	**1.1. Is the qualitative approach appropriate to answer the research question?** *Yes (gather opinions about proposed service)*<br>**1.2. Are the qualitative data collection methods adequate to address the research question?** *Yes (focus groups and interviews with different staff groups at different sites)*<br>**1.3. Are the findings adequately derived from the data?** *Yes*<br>**1.4. Is the interpretation of results sufficiently substantiated by data?** *Yes (supported by relevant quotes)*<br>**1.5. Is there coherence between qualitative data sources, collection, analysis and interpretation?** *Yes (supported by use of Theoretical Domains Framework)*
Madden 2022[14]	**S1. Are there clear research questions?** *Yes*<br><br>**S2. Do the collected data allow to address the research questions?** *Yes*	Qualitative	**1.1. Is the qualitative approach appropriate to answer the research question?** *Yes (pharmacists' experience of SMR implementation)*<br>**1.2. Are the qualitative data collection methods adequate to address the research question?** *Yes (interviews with newly employed and established pharmacists)*<br>**1.3. Are the findings adequately derived from the data?** *Yes*<br>**1.4. Is the interpretation of results sufficiently substantiated by data?** *Yes (supported by relevant quotes)*<br>**1.5. Is there coherence between qualitative data sources, collection, analysis and interpretation?** *Yes (supported by thematic analysis)*
Peek 2020[15]	**S1. Are there clear research questions?** *Yes*	Quantitative non-randomised	**3.1. Are the participants representative of the target population?** *Yes (general practices and their patients)*<br>**3.2. Are measurements appropriate regarding both the outcome and intervention (or exposure)?** *Can't tell (for intervention)*

	**S2. Do the collected data allow to address the research questions?** *Yes*		**3.3. Are there complete outcome data?** *Yes*<br>**3.4. Are the confounders accounted for in the design and analysis?** *No (small risk of unmeasured confounding)*<br>**3.5. During the study period, is the intervention administered (or exposure occurred) as intended?** *Can't tell (interventions not externally validated)*
Rodgers 2022[16]	**S1. Are there clear research questions?** *Yes*<br><br>**S2. Do the collected data allow to address the research questions?** *Yes*	Quantitative non-randomised	**3.1. Are the participants representative of the target population?** *Yes (general practices and their patients)*<br>**3.2. Are measurements appropriate regarding both the outcome and intervention (or exposure)?** *Can't tell (for intervention)*<br>**3.3. Are there complete outcome data?** *No (6- and 12-month data not collected from all practices)*<br>**3.4. Are the confounders accounted for in the design and analysis?** *No (small risk of unmeasured confounding)*<br>**3.5. During the study period, is the intervention administered (or exposure occurred) as intended?** *Can't tell (interventions not externally validated)*
Syafhan 2021[17]	**S1. Are there clear research questions?** *Yes*<br><br>**S2. Do the collected data allow to address the research questions?** *Yes*	Quantitative randomised controlled trial	**2.1. Is randomisation appropriately performed?** *Can't tell (method of randomisation not reported)*<br>**2.2. Are the groups comparable at baseline?** *Yes*<br>**2.3. Are there complete outcome data?** *No (30% lost to follow-up or withdrew)*<br>**2.4. Are outcome assessors blinded to the intervention provided?** *Can't tell (outcome data from GP electronic records)*<br>**2.5 Did the participants adhere to the assigned intervention?** *No (30% lost to follow-up or withdrew)*
Thayer 2021[23]	**S1. Are there clear research questions?** *Yes*<br><br>**S2. Do the collected data allow to address the research questions?** *Yes*	Quantitative non-randomised	**3.1. Are the participants representative of the target population?** *Yes (care home residents with intellectual disabilities)*<br>**3.2. Are measurements appropriate regarding both the outcome and intervention (or exposure)?** *Yes (details recorded for each review and associated outcomes)*<br>**3.3. Are there complete outcome data?** *Yes (all specified outcomes reported)*<br>**3.4. Are the confounders accounted for in the design and analysis?** *No (uncontrolled before/after study)*

			**3.5. During the study period, is the intervention administered (or exposure occurred) as intended?** *Yes (one-off review mainly based on records)*
Twigg 2015[24]	**S1. Are there clear research questions?** *Yes*<br><br>**S2. Do the collected data allow to address the research questions?** *Yes*	Quantitative non-randomised	**3.1. Are the participants representative of the target population?** *Can't tell (no indication of attempts to recruit a representative sample)*<br>**3.2. Are measurements appropriate regarding both the outcome and intervention (or exposure)?** *Yes (details recorded for intervention components and associated outcomes)*<br>**3.3. Are there complete outcome data?** *Can't tell (limited response for resource use outcomes)*<br>**3.4. Are the confounders accounted for in the design and analysis?** *No (uncontrolled before/after study)*<br>**3.5. During the study period, is the intervention administered (or exposure occurred) as intended?** *Can't tell (approx. 30% withdrawal rate)*
