# Supplementary material for: Pharmacist-led primary care interventions to promote medicines optimisation and reduce overprescribing: a systematic review of UK studies and initiatives
Source: BMJ Open. 2024 Aug 7;14(8):e081934. doi: 10.1136/bmjopen-2023-081934 (PMC11407218; doi:10.1136/bmjopen-2023-081934)
Supplement: Supplementary file 4 [file bmjopen-2023-081934.reviewer_comments.pdf]

PEER REVIEW HISTORY

BMJ Open publishes all reviews undertaken for accepted manuscripts. Reviewers are asked to complete a checklist review form (**http://bmjopen.bmj.com/site/about/resources/checklist.pdf**) and are provided with free text boxes to elaborate on their assessment. These free text comments are reproduced below.

ARTICLE DETAILS

**TITLE (PROVISIONAL)**	Pharmacist-led primary care interventions to promote medicines optimisation and reduce overprescribing: a systematic review of UK studies and initiatives
**AUTHORS**	Chambers, Duncan; Preston, Louise; Clowes, Mark; Cantrell, Anna; Goyder, Elizabeth

VERSION 1 – REVIEW

**REVIEWER**	Bell, Brian<br>University of Nottingham
**REVIEW RETURNED**	25-Jan-2024

**GENERAL COMMENTS**	My comments are fairly minor. First, in the appendices, there are search terms listed for PsychInfo, yet no mention is made of PsychInfo on page 8, did the authors search PsychInfo, and if so, what were the results of that search? Second, with regard to the searches, the authors state that they searched Cinahl, but again, with respect to the Appendix, I believe the Ebscohost entry on page 88 lists the Cinahl search terms, but it's not labeled as the Cinahl search so I wasn't quite clear about that. There are several places throughout the manuscript where abbreviations are used but not spelled out so that was a little hard to figure out. For example, on page 18, the authors say'... involving care home residents with ID'. I know this refers to intellecutal disabilities, but the authors should spell this out. Also, I know that SMR refers to structured medication review, but the authors should spell this out when the reader encounters the first instance of it. The authors should also note that a systematic review of pharmacist led interventions to optimise prescribing was conducted by Riordan (2016). They should probably cite this as that review was up to 2015 and doesn't represent much overlap with the current review.

**REVIEWER**	Poluektova, Olga<br>Economic and Social Research Institute
**REVIEW RETURNED**	13-Feb-2024

**GENERAL COMMENTS**	The paper entitled "Pharmacist-led primary care interventions to promote medicines optimisation and reduce overprescribing: a systematic review of UK studies and initiatives" by Chambers et al. reviews studies reporting on pharmacist-led medication review interventions to assess their effectiveness, identify the main characteristics of such interventions and potential barriers and facilitators to their implementation. The authors conclude that such interventions seem mostly effective, although the results should be interpreted cautiously, given the predominantly non-experimental evidence in the review. Overall, the review addresses an important

	topic and the authors have done a good job. However, it could benefit from a more expansive discussion on certain aspects and the rationale behind methodological choices.<br><br>1. The introduction could be a bit more focused. I would bring the review's aim closer to the beginning and be more specific about what types of interventions you are reviewing, what exactly they involve and what they try to achieve. On the one hand, you seem to set the focus on situations when initial prescription has already been done, the intervention of interest is medication review by pharmacist and the outcome of interest is deprescribing (the second paragraph of the introduction), but on the other, you seem rather vague in the paragraph where you state the aim of the review with regards to the outcomes of interest (i.e., you say "outcomes related to prescribing"). In addition, you seem to have used "shared decision-making" and "personalised care" as search terms, suggesting a broader focus of the interventions you have included.<br><br>2. Currently, it is unclear why you focus exclusively on pharmacist-led interventions; you need a stronger justification here. In the introduction, you mention that doctors primarily order prescriptions (p. 5, lines 47-48), so why exclude physician-led medication review interventions?<br><br>3. You say that your objectives were to "assess the effectiveness of interventions, identify their key characteristics" and "the main barriers and facilitators to implementation". However, you do not mention barriers and facilitators among your inclusion criteria, nor are they present in the table summarising your search strategy (on p.8) for the first objective. Additionally, you only state one objective (evaluating the effectiveness) in the abstract, arguably the least achievable by your review, given the designs of the studies you reviewed.<br><br>4. The "Review aims and objectives" section in the Method section might be better suited for the Introduction section.<br><br>5. On p. 8 (lines 24-27), you say that you searched for studies published in OECD countries with healthcare systems similar to UK. My understanding was that your focus was on UK studies only, haven't you only searched for research conducted in the UK?<br><br>6. On p. 8 in "Study Selection" you mention that disagreements were resolved by discussion. What was the level of disagreement between the researchers involved in screening of studies?<br><br>7. In the table with the studies in your review (p. 13), I would group studies by design (from stronger to less strong) and not in alphabetic order.<br><br>8. Given the quality of the studies you had reviewed, I do not think your main conclusion should be that pharmacist-led interventions effectively reduce overprescription (as it appears after reading the abstract). It seems that the existing research does not allow you to conclude with certainty that the improvement in the outcomes can be attributed to the intervention, given that most studies you reviewed did not use experimental designs with a control group. Rather, it is apparent that there is a need for more high-quality studies using stronger designs to be able to make conclusions

	about the effectiveness of pharmacist-led interventions. This seems like a more important point to make, so I would suggest building your discussion around this point and around the barriers and how they can be addressed.<br><br>9. When you talk about the implications for research (p.30), you say that “qualitative research is needed better to understand patient and family attitudes to shared decision making”. It is not really the methodology question; you could also study this issue using quantitative research methods. You can omit “qualitative” and just say more research focusing on patients’ attitudes is needed, and perhaps not just attitudes but also their awareness of the issue of polypharmacy and problems associated with it.<br><br>10. In Conclusions, it is not entirely clear what you mean by “Although not a focus of this review, there appears to be a shortage of high-quality economic evidence to guide decision-making.” (p. 31-32). You mean studies on decision-making among prescribers?

VERSION 1 – AUTHOR RESPONSE

Reviewer: 1
Dr. Brian Bell, University of Nottingham
Comments to the Author:
My comments are fairly minor. First, in the appendices, there are search terms listed for PsychInfo, yet no mention is made of PsychInfo on page 8, did the authors search PsychInfo, and if so, what were the results of that search? Second, with regard to the searches, the authors state that they searched Cinahl, but again, with respect to the Appendix, I believe the Ebscohost entry on page 88 lists the Cinahl search terms, but it's not labeled as the Cinahl search so I wasn't quite clear about that. There are several places throughout the manuscript where abbreviations are used but not spelled out so that was a little hard to figure out. For example, on page 18, the authors say'... involving care home residents with ID'. I know this refers to intellecutal disabilities, but the authors should spell this out. Also, I know that SMR refers to structured medication review, but the authors should spell this out when the reader encounters the first instance of it. The authors should also note that a systematic review of pharmacist led interventions to optimise prescribing was conducted by Riordan (2016). They should probably cite this as that review was up to 2015 and doesn't represent much overlap with the current review.

Response
Yes, we did search PsycINFO and CINAHL – retrieving 44 and 307 results respectively. We have amended the abstract and methods sections accordingly. The search strategies are those reproduced in the appendix, though for layout reasons the database name was removed from all but the final line of the EBSCO search. The methods section of the text was based on an earlier version of the protocol which did not include these additional sources. We apologise for this omission.
We have replaced ‘IDs’ by ‘intellectual disabilities’ throughout and spelled out SMR at its first occurrence in text and tables.
We have cited and discussed the review by Riordan et al. in the Discussion (p28).

Reviewer: 2
Dr. Olga Poluektova, Economic and Social Research Institute
Comments to the Author:

The paper entitled “Pharmacist-led primary care interventions to promote medicines optimisation and reduce overprescribing: a systematic review of UK studies and initiatives” by Chambers et al. reviews studies reporting on pharmacist-led medication review interventions to assess their effectiveness, identify the main characteristics of such interventions and potential barriers and facilitators to their implementation. The authors conclude that such interventions seem mostly effective, although the results should be interpreted cautiously, given the predominantly non-experimental evidence in the review. Overall, the review addresses an important topic and the authors have done a good job. However, it could benefit from a more expansive discussion on certain aspects and the rationale behind methodological choices.

1. The introduction could be a bit more focused. I would bring the review's aim closer to the beginning and be more specific about what types of interventions you are reviewing, what exactly they involve and what they try to achieve. On the one hand, you seem to set the focus on situations when initial prescription has already been done, the intervention of interest is medication review by pharmacist and the outcome of interest is deprescribing (the second paragraph of the introduction), but on the other, you seem rather vague in the paragraph where you state the aim of the review with regards to the outcomes of interest (i.e., you say “outcomes related to prescribing”). In addition, you seem to have used “shared decision-making” and “personalised care” as search terms, suggesting a broader focus of the interventions you have included.
Response: Thank you; we have revised the introduction to introduce the review and its context earlier (paragraph 1). The focus is on medication review after prescription but this involves sahred decision making about whether or not to make any changes

2. Currently, it is unclear why you focus exclusively on pharmacist-led interventions; you need a stronger justification here. In the introduction, you mention that doctors primarily order prescriptions (p. 5, lines 47-48), so why exclude physician-led medication review interventions?
Response: The review was done by members of the Sheffield HS&DR Evidence Synthesis Centre, which provides a responsive service to review topics prioritised by the NIHR HS&DR programme in response to requests from key stakeholders. The request for a review focusing specifically on pharmacist-led interventions originated from NHS England’s National Clinical Director for Prescribing and is intended to support implementation of the findings of the National Overprescribing Review for England as mentioned in the final paragraph of the Introduction.

3. You say that your objectives were to “assess the effectiveness of interventions, identify their key characteristics” and “the main barriers and facilitators to implementation”. However, you do not mention barriers and facilitators among your inclusion criteria, nor are they present in the table summarising your search strategy (on p.8) for the first objective. Additionally, you only state one objective (evaluating the effectiveness) in the abstract, arguably the least achievable by your review, given the designs of the studies you reviewed.
Response: Our aims and objectives included ‘identification of case study examples of effectively implemented interventions in the UK’. Papers reporting on implementation were selected from the results of the literature search which was deliberately broad. We have added ‘barriers and facilitators to implementation’ to the objectives in the abstract.

4. The “Review aims and objectives” section in the Method section might be better suited for the Introduction section.
Response: On balance we feel the numbered list of components fits better into the methods, although we don’t feel strongly about this.

5. On p. 8 (lines 24-27), you say that you searched for studies published in OECD countries with healthcare systems similar to UK. My understanding was that your focus was on UK studies only, haven’t you only searched for research conducted in the UK?

Response: A single literature search covering the UK and other OECD countries was done for both this review and an associated scoping review with a broader focus (Search methods, line 1). For this review, only UK studies were selected.

6. On p. 8 in “Study Selection” you mention that disagreements were resolved by discussion. What was the level of disagreement between the researchers involved in screening of studies?
Response: We added the following in the text: A good level of agreement was achieved, values of kappa between pairs of reviewers ranging from 0.67 to 0.96.

7. In the table with the studies in your review (p. 13), I would group studies by design (from stronger to less strong) and not in alphabetic order.
Response: We have revised Table 1 as suggested.

8. Given the quality of the studies you had reviewed, I do not think your main conclusion should be that pharmacist-led interventions effectively reduce overprescription (as it appears after reading the abstract). It seems that the existing research does not allow you to conclude with certainty that the improvement in the outcomes can be attributed to the intervention, given that most studies you reviewed did not use experimental designs with a control group. Rather, it is apparent that there is a need for more high-quality studies using stronger designs to be able to make conclusions about the effectiveness of pharmacist-led interventions. This seems like a more important point to make, so I would suggest building your discussion around this point and around the barriers and how they can be addressed.
Response: We believe the underlying structure of the discussion is valid but have revised the abstract and discussion to reflect a more cautious interpretation of the findings.

9. When you talk about the implications for research (p.30), you say that “qualitative research is needed better to understand patient and family attitudes to shared decision making”. It is not really the methodology question; you could also study this issue using quantitative research methods. You can omit “qualitative” and just say more research focusing on patients’ attitudes is needed, and perhaps not just attitudes but also their awareness of the issue of polypharmacy and problems associated with it.
Response: We agree and have deleted ‘qualitative’.

10. In Conclusions, it is not entirely clear what you mean by “Although not a focus of this review, there appears to be a shortage of high-quality economic evidence to guide decision-making.” (p. 31-32). You mean studies on decision-making among prescribers?
Response: We meant decision-making by service providers and commissioners and have added this in.

VERSION 2 – REVIEW

**REVIEWER**	Poluektova, Olga<br>Economic and Social Research Institute
**REVIEW RETURNED**	07-May-2024

**GENERAL COMMENTS**	I appreciate the authors' efforts in clarifying and refining the manuscript; the paper now appears clearer and more focused. However, I believe further explanation is needed in the Introduction regarding the rationale behind the focus on pharmacist-led interventions. While I understand that the study was commissioned and conducted in response to the requests and priorities of key stakeholders, providing a more detailed justification would enhance the paper and make its findings more interesting to a broader audience.

	Referring to previous research, as the authors do in the Discussion (subsection “Relationship to previous research”), could further strengthen the foundation of the study. I suggest including a brief overview of current research in the Introduction, along with a state-of-the-art analysis. This addition would help readers better understand the contribution of the study and its significance within the existing research.
